# Increased Antimicrobial Consumption, Isolation Rate, and Resistance Profiles of Multi-Drug Resistant *Klebsiella pneumoniae*, *Pseudomonas aeruginosa,* and *Acinetobacter baumannii* During the COVID-19 Pandemic in a Tertiary Healthcare Institution

**DOI:** 10.3390/antibiotics14090871

**Published:** 2025-08-29

**Authors:** Predrag Savic, Ljiljana Gojkovic Bukarica, Predrag Stevanovic, Teodora Vitorovic, Zoran Bukumiric, Olivera Vucicevic, Nenad Milanov, Vladimir Zivanovic, Ana Bukarica, Milos Gostimirovic

**Affiliations:** 1 University Hospital Centre Dr Dragisa Misovic–Dedinje, 11000 Belgrade, Serbia; predrag.savic@dragisamisovic.bg.ac.rs (P.S.); bukarica@rcub.bg.ac.rs (L.G.B.); predrag.stevanovic@med.bg.ac.rs (P.S.); teodora.vitorovic@zastitazdravlja.rs (T.V.); olivera.vucicevic@dragisamisovic.bg.ac.rs (O.V.); nenad.milanov@dragisamisovic.bg.ac.rs (N.M.); vladimir.zivanovic@dragisamisovic.bg.ac.rs (V.Z.); 2 Institute of Pharmacology, Clinical Pharmacology and Toxicology, Faculty of Medicine, University of Belgrade, 11000 Belgrade, Serbia; 3 Institute of Statistics and Informatics, Faculty of Medicine, University of Belgrade, 11000 Belgrade, Serbia; zoran.bukumiric@med.bg.ac.rs; 4 Institute for Cardiovascular Diseases Dedinje, University of Belgrade, 11000 Belgrade, Serbia; ana.bukarica@institutdedinje.org

**Keywords:** COVID-19 infection, antibiotic consumption, MDR bacterial isolates, antibiotic resistance

## Abstract

Background: The aims of this paper are to examine the impact of the COVID-19 pandemic on the non-rational use of antibiotics and potential alterations in the antibiotic resistance profiles of multi-drug resistant (MDR) isolates of *Klebsiella pneumoniae* (KPN), *Pseudomonas aeruginosa* (PAE), and *Acinetobacter baumannii* (ABA). Material and Methods: This study was conducted at the tertiary University Hospital “Dr Dragisa Misovic-Dedinje” (Belgrade, Serbia) and was divided into three periods: pre-pandemic (1.4.2019–31.3.2020, period I), COVID-19 pandemic (1.4.2020–31.3.2021, period II), and COVID-19 pandemic-second phase (1.4.2021–31.3.2022, period III). Cultures were taken from each patient with clinically suspected infection (symptoms, biochemical markers of infection). All departments of the hospital were included in this study. Based on the source, all microbiological specimens were divided into 1° blood, 2° respiratory tract (tracheal aspirate, bronchoalveolar lavage fluid, throat, sputum), 3° central-line catheter, 4° urine, 5° urinary catheter, 6° skin and soft tissue, and 6° other (peritoneal fluid, drainage sample, bioptate, bile, incisions, fistulas, and abscesses). After the isolation of bacterial strains from the samples, an antibiotic sensitivity test was performed for each individual isolate with the automated Vitek^®^ 2 COMPACT. Antibiotic consumption testing was performed by the WHO guideline equations (ATC/DDD). Results: A total of 2196 strains of KPN, PAE, and ABA from 41,144 hospitalized patients were isolated (23.6% of the number of total isolates). The number of ABA isolates statistically increased (*p* = 0.021), while the number of PAE isolates statistically decreased (*p* = 0.003) during the pandemic. An increase in the percentage of MDR strains was observed for KPN (*p* = 0.028) and PAE (*p* = 0.027). There has been an increase in the antibiotic resistance of KPN for piperacillin–tazobactam, the third and fourth generations of cephalosporins (ceftriaxone, ceftazidime, and cefepime), all carbapanems (imipenem, meropenem, and ertapenem), and levofloxacin; of PAE for imipenem; and of ABA for amikacin. Total antibiotic consumption increased (from 755 DBD to 1300 DBD, +72%), especially in the watch and reserve group of antibiotics. The highest increases were noted for vancomycin, levofloxacin, azithromycin, and meropenem. MV positively correlated with the increased occurrence of MDR KPN (r = 0.35, *p* = 0.009) and MDR PAE (r = 0.43, *p* = 0.009) but not for MDR ABA (r = 0.09, *p* = 0.614). There has been a statistically significant increase in the *Candida* sp. isolates, but the prevalence of *Clostridium difficile* infection remained unchanged. Conclusions: The COVID-19 pandemic has influenced the increase in total and MDR strains of KPN, ABA, and PAE and worsened their antibiotic resistance profiles. An increase in the consumption of both total and specific antibiotics was observed, mostly of fluoroquinolones and carbapenems. A positive correlation between the number of patients on MV and an increase in MDR KPN and MDR PAE strains was noted. It is necessary to adopt and demand the implementation of appropriate antimicrobial stewardship interventions to decrease the resistance of intrahospital pathogens to antibiotics.

## 1. Introduction

By 28 July 2024, the COVID-19 pandemic had affected around 775 million people worldwide and caused nearly 7 million deaths [1]. Countries around the world have suffered global socioeconomic losses due to serious disruptions in the organization of health systems and high mortality caused by the multi-factorial impact of the virus. The most serious indirect impact of the COVID-19 pandemic in hospital settings may be the emergence of a “silent” pandemic of bacterial nosocomial infections caused by pathogens that have developed a greater degree of resistance to antimicrobial drugs (AMRs), especially in critically ill patients in the intensive care units (ICUs) [2]. The most common multi-drug-resistant (MDR) bacteria associated with significant global mortality from nosocomial (hospital-acquired) infections (HAIs) are *Klebsiella pneumoniae* (KPN), *Acinetobacter baumannii* (ABA), and *Pseudomonas aeruginosa* (PAE), and their impact on mortality during the COVID-19 pandemic cannot be neglected [3].

It is estimated that resistance to antimicrobial drugs caused more than 1.2 million deaths in 2019 and might cause approximately 10 million per year by 2050 [4]. To combat antimicrobial resistance, guidelines suggest limiting the use of antibiotics and using them exclusively for proven bacterial infections. However, referring to some experiences from the past, the first-world protocols for the treatment of COVID–19 infection also included antibiotics, most often azithromycin. Indeed, the consumption of azithromycin in March 2020 was 400% greater than that in February 2020, as reported in Spain [5]. Similarly, in the National Recommendations for the treatment of COVID–19 infection in Serbia at the beginning of 2020 (versions 1–3), it was advised to apply antibiotics empirically due to possible bacterial co-infection associated with viral pneumonia; therefore, combination therapy with azithromycin and levofloxacin was recommended [6]. The duration of treatment ranged from 5 to 7 days depending on the severity of the disease and whether the patient was receiving combined therapy comprising a macrolide and a fluoroquinolone or a macrolide or a cephalosporin. This choice of antibiotics was based on previous knowledge that the influenza virus is often associated with bacterial coinfections [7,8]. Bearing this in mind, the fear of this outcome for COVID-19 infection caused complete uncritical use of antibiotics at the beginning of 2020 in all those infected with the SARS-CoV-2 virus, regardless of whether a bacterial infection was proven. Several studies conducted in 2020 showed that approximately 70% of patients with COVID–19 infection received antibiotics, and 100% of those patients did so in the ICU [9]. However, other studies have shown that only 3–8% of patients suffering from COVID-19 infection actually have proven bacterial or fungal co-infection [10]. Van Laethem et al. (2021) showed that 4.7% of hospitalized patients with COVID-19 had hospital-acquired bacterial superinfection, while 14.6% had this type of infection in the ICUs [11].

Even before the COVID-19 pandemic, the consumption of antibiotics in Serbia was much higher than the European average, as evidenced by the ten-year trend analysis, by which broad-spectrum macrolides, fluoroquinolones, and third-generation cephalosporins show the greatest increase [12]. These factors may have impacted the increased resistance of common G-negative pathogens to antibiotics.

Previously, we reported an alarming increase in the resistance of KPN and ABA at the University Hospital Centre “Dr. Dragisa Misovic-Dedinje” (Belgrade, Serbia) during the 2012–2015 period [13]. The increased prevalence was explained by unrestricted antibiotic use and irrational prescribing practices, addressing the need for further evaluation of prescribing rates and the percentages of patients with MDR and antimicrobial resistance (AMR). In the years that followed, no factor has impacted the increase in MDR bacteria or the decrease in bacterial sensitivity to antibiotics as a result of the recent COVID-19 pandemic. This coinfection certainly affects the complications and prolongs the hospitalization of critically ill COVID-19 patients. Finally, sepsis, respiratory distress, multisystem organ failure, and respiratory insufficiency may develop, which require intubation and mechanical ventilation (MV). These findings urged the need for updated studies of MDR/AMR surveillance to reduce mortality in patients, to assess the damage caused to health systems, and to define a strategy to control the newly emerging bacterial resistance that is present not only in hospitals worldwide but also in Serbia.

For these purposes, the aims of this paper are as follows:

1. To investigate whether the COVID-19 pandemic has influenced the antibiotic consumption;
2. To investigate the influence of the COVID-19 pandemic on the prevalence of the total number and MDR isolates of KPN, ABA, and PAE;
3. To test whether there was a change in antimicrobial resistance before and during the COVID-19 pandemic;
4. To investigate whether the number of patients on MV has influenced the occurrence of MDR pathogens and antibiotic consumption.

## 2. Results

### 2.1. Characteristics of Patients, Prescribed Antimicrobial Therapy, and Bacterial Isolates

The number of patients (total, those in the ICU, those on MV) and the average duration of hospital stay (total and of the patients in the ICU) are shown in Table 1. In all three study periods, a total of 41,144 patients were admitted in the hospital. There was a statistically significant decrease in the number of admitted patients in periods II and III compared to period I (*p* < 0.001, both). There was a statistically significant decrease in the number of patients in the ICU in periods II and III compared to period I (*p* < 0.001, both). There was a statistically significant increase in the number of patients in the ICU who are on MV in period II (72.4%, *p* = 0.002) but not in period III (36.4%, *p* > 0.05) compared to period I (10.7%). Regarding total hospital admissions, the average duration of hospital stays increased in period II (*p* < 0.05) but not in period III (*p* > 0.05), compared to period I. On the contrary, in periods I and III, the average duration of hospital stay in the ICU was longer than the average duration of hospital stay in the ICU in period II (*p* < 0.05).

**Figure 1:**
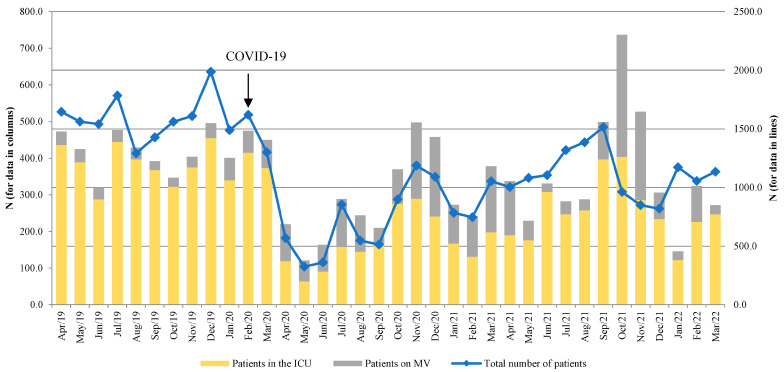
The distribution of total number of patients, patients in the ICU, and patients on mechanical ventilation (MV) by month. Left axis—total number of patients, right axis—patients in the ICU and patients on MV.

The monthly distributions of the total number of patients, patients in the ICU, and the number of patients on MV are shown in Figure 1. An increasing trend in the number of admitted patients during the delta viral variant (June 2021) was observed.

The main indications for antibiotic therapy during the whole interval of study are summarized in Table 2. During period I, respiratory tract infections, urinary tract infections, and skin and soft tissue infections were the most common diagnoses. During period II, the three most common diagnoses were respiratory tract infections, COVID-19 infection, and urinary tract infections. During period III, the three most common diagnoses were COVID-19 infection, respiratory tract infections, and urinary tract infections. The percentage of patients with *C. difficile* infection relative to the total number of indications for antimicrobial therapy was greater in period III than in periods I and II (*p* < 0.05). During this study, there was an increase in the total number of indications for antibiotic therapy (*p* < 0.05).

A total of 9246 isolates were detected in a three-year interval. The most common strains isolated during period I were *E. coli* (18.2%), *Enterococcus* spp. (11.1%), and *KPN* (9.5%). During period II, the most common isolates were *ABA* (12.9%), *Enterococcus* spp. (11.2%), and *KPN* (9.6%). During period III, the most prevalent isolates were *Enterococcus* spp. (15.2%), *E. coli* (11.6%), and *KPN* (11.8%) (Table 3). In period II, there was a statistically significant decrease in the number of total isolates of *E. coli*, *PAE*, *P. mirabilis*, *S. aureus*, *Enterobacter* sp., and *Streptococcus* sp. and a statistically significant increase in the number of total isolates of *ABA*, *S. epidermidis*, *E. faecium,* and *Candida* sp. (*p* < 0.05, for all). In period III, there was a statistically significant decrease in the number of total isolates of *PAE*, *E. faecalis*, *Enterobacter* sp., and *Streptococcus* sp. and a statistically significant increase in the number of total isolates of *Candida* sp. (*p* < 0.05, for all).

### 2.2. Distribution of Total Number and MDR Isolates of KPN, ABA, and PAE by Years and Months

**Figure 2:**
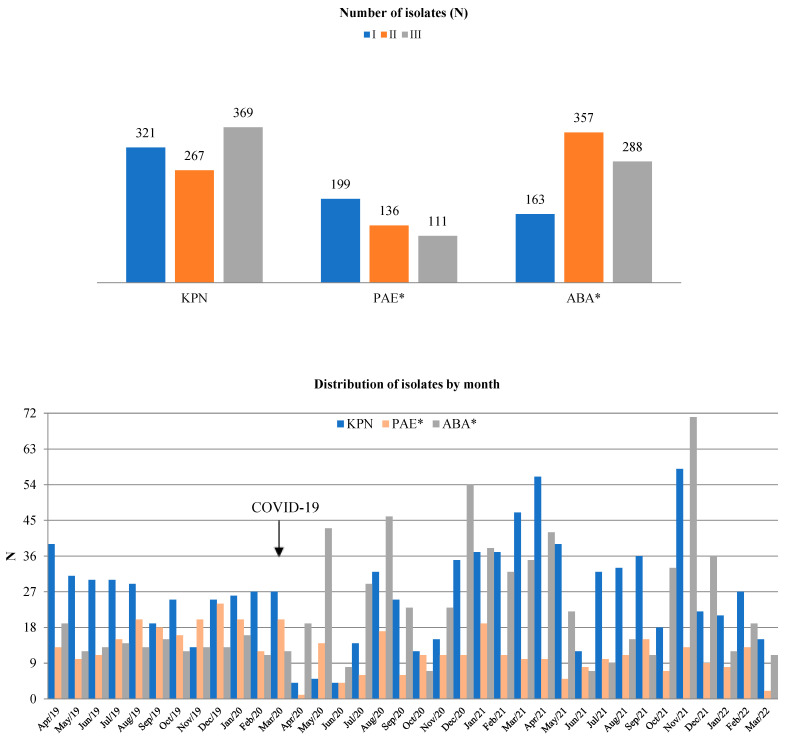
The total number of KPN, PAE, and ABA isolates by study year (**top**) and by study month (**bottom**). *—*p* < 0.05. There was a statistically significant decrease in the number of total PAE isolates in periods II and III compared to period I and a statistically significant increase in the number of ABA isolates in period II.

The distributions of KPN, ABA, and PAE isolates based on the site of infection and specimen type are presented in Table 4. The annual numbers of isolated KPN, ABA, and PAE strains and their distributions by month are shown in Figure 2. Out of all the isolates, the total numbers of KPNs were 321/3358 (9.5%), 267/2772 (9.6%), and 369/3116 (11.8%) in periods I, II, and III, respectively. During the same period, the total numbers of PAE isolates were 199/3358 (5.9%), 121/2772 (4.4%), and 111/3116 (3.6%), while the total numbers of ABA isolates were 163/3358 (4.9%), 357/2772 (12.9%), and 288/3116 (9.2%). Compared to period I, there was a statistically significant increase in the total number of ABA isolates in period II (*p* = 0.021) and a statistically significant decrease in the total number of PAE isolates in period II (*p* = 0.003) and period III (*p* = 0.001, Figure 2). The total number of KPN isolates did not significantly change throughout the study periods (F = 1.33, *p* = 0.278).

**Figure 3:**
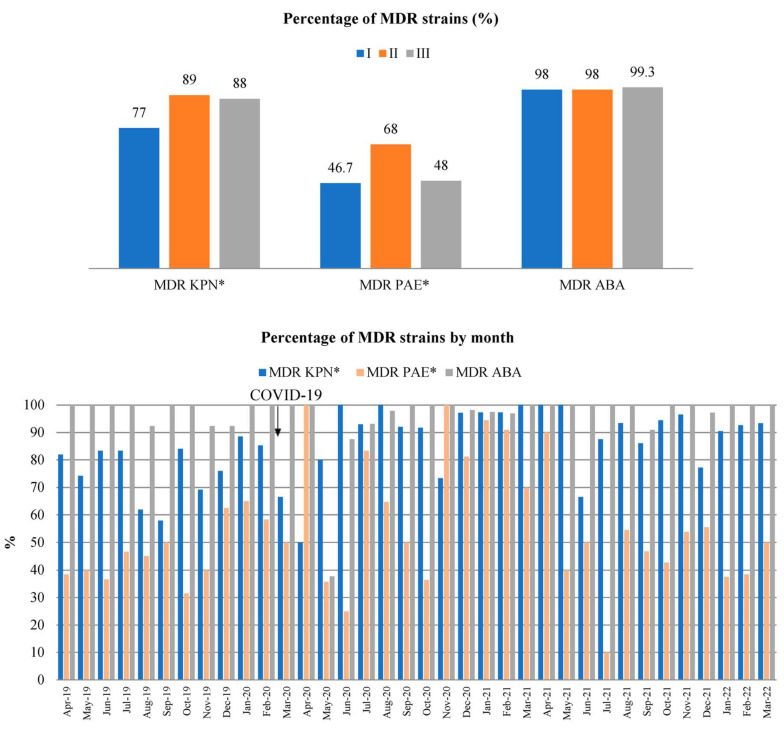
The percentage of MDR KPN, PAE, and ABA strains by study year (**top**) and study month (**bottom**). *—*p* < 0.05. There was a statistically significant increase in the percentage of MDR KPN in periods II and III compared to period I. The percentage of MDR strains is calculated as the proportion of MDR isolates out of the total number of KPN, PAE, and ABA isolates.

The percentages of MDR KPN, PAE, and ABA strains in relation to the total number of KPN, PAE, and ABA isolates per year and their distribution by month for the study periods are shown in Figure 3.

The percentages of MDR KPN during periods I, II, and III were 77% (247/321), 89% (237/267), and 88% (324/369), respectively. The percentages of MDR PAE during study periods I, II, and III were 47% (93/199), 68% (82/121), and 48% (53/111), respectively. The percentages of MDR ABA during periods I, II, and III were 98% (160/163), 98% (349/357), and 99% (286/288), respectively.

Compared to period I, there was a statistically significant increase in the percentage of MDR KPN in periods II (*p* = 0.028) and III (*p* = 0.021). Compared to the same period, there was a statistically significant increase in the percentage of MDR PAE in period II (*p* = 0.027), after which a statistically significant decrease in MDR PAE was observed (*p* = 0.031).

The percentage of MDR ABA isolates did not significantly change throughout study periods (F = 1.41, *p* = 0.259).

A detailed overview of the statistically significant changes in the number of total and MDR isolates of KPN, PAE, and ABA is shown in Supplementary File S1.

### 2.3. Profiles of Antibiotic Resistance in KPN, PAE, and ABA Isolates

(A) KPN

A statistically significant increase in the resistance of KPN in period II was noted for piperacillin–tazobactam (87.76 ± 14.38% (CI: 78.62–96.89) in period II vs. 71.63 ± 10.72% (CI: 64.81–78.44) in period I, *p* = 0.011), ceftriaxone (89.73 ± 17.82% (CI: 78.41–101.06) in period II vs. 76.08 ± 9.86% (CI: 69.82–82.35) in period I, *p* = 0.039), ceftazidime (89.87 ± 14.68% (CI: 80.54–99.19) in period II vs. 75.33 ± 10.14% (CI: 68.89–81.77) in period I, *p* = 0.008), cefepime (88.82 ± 14.16% (CI: 79.82–97.82) in period II vs. 71.73 ± 11.33% (CI: 64.54–78.93) in period I, *p* = 0.009), imipenem (46.65 ± 32.66% (CI: 25.9–67.4) in period II vs. 20.28 ± 10.07% (CI: 13.88–26.68) in period I, *p* =0.034), meropenem (69.12 ± 24.94% (CI: 53.27–84.96) in period II vs. 35.64 ± 15.68% (CI: 25.68–45.6) in period I, *p* = 0.003), ertapenem (83 ± 20.39% (CI: 70.04–95.96) in period II vs. 58.33 ± 16.56% (CI: 47.81–68.86) in period I, *p* = 0.005), and levofloxacin (91.09 ± 15.17% (CI: 81.45–100.73) in period II vs. 78.04 ± 8.17% (CI: 72.85–83.24) in period I, *p* = 0.047).

A statistically significant increase in the resistance of KPN in period III was noted for piperacillin–tazobactam (84.87 ± 12.48% (CI: 76.93–92.8) in period III vs. 71.63 ± 10.72% (CI: 64.81–78.44) in period I, *p* = 0.045), ceftriaxone (89.83 ± 8.41% (CI: 84.48–95.17) in period III vs. 76.08 ± 9.86% (CI: 69.82–82.35) in period I, *p* = 0.037), ceftazidime (89.95 ± 6.81% (CI: 85.62–94.28) in period III vs. 75.33 ± 10.14% (CI: 68.89–81.77) in period I, *p* = 0.008), cefepime (85.98 ± 13.67% (CI: 77.3–94.67) in period III vs. 71.73 ± 11.33% (CI: 64.54–78.93) in period I, *p* = 0.036), imipenem (55.24 ± 24.01% (CI: 39.99–70.49) in period III vs. 20.28 ± 10.07% (CI: 13.88–26.68) in period I, *p* =0.004), meropenem (65.17 ± 25.38% (CI: 49.04–81.29) in period III vs. 35.64 ± 15.68% (CI: 25.68–45.6) in period I, *p* = 0.033), and ertapenem (79.02 ± 15.25% (CI: 69.33–88.72) in period II vs. 58.33 ± 16.56% (CI: 47.81–68.86) in period I, *p* = 0.02).

There was no difference in the antibiotic resistance of KPN between periods III and II.

(B) PAE

A statistically significant increase in the resistance of PAE in period II was noted for imipenem (67.07 ± 26.74% (CI: 50.08–84.06) in period II vs. 39.5 ± 10.1% (CI: 33.09–45.91) in period I, *p* = 0.004).

A statistically significant decrease in the resistance of PAE in period III was noted for imipenem (45.83 ± 17.27% (CI: 34.85–56.8) in period III vs. 67.07 ± 26.74% (CI: 50.08–84.06) in period II, *p* = 0.033) and ciprofloxacin (43.54 ± 20.42% (CI: 30.57–56.52) in period III vs. 66.27 ± 26.65% (CI: 49.33–83.2) in period II, *p* = 0.041).

(C) ABA

A statistically significant increase in the resistance of ABA in period II was noted for amikacin (83.03 ± 13.4% (CI: 74.52–91.55) in period II vs. 66.58 ± 13.9% (CI: 57.75–75.41) in period I, *p* = 0.015) as well as in period III (80.29 ± 12.72% (CI: 72.21–88.38) in period III vs. 66.58 ± 13.9% (CI: 57.75–75.41) in period I, *p* = 0.051).

**Figure 4:**
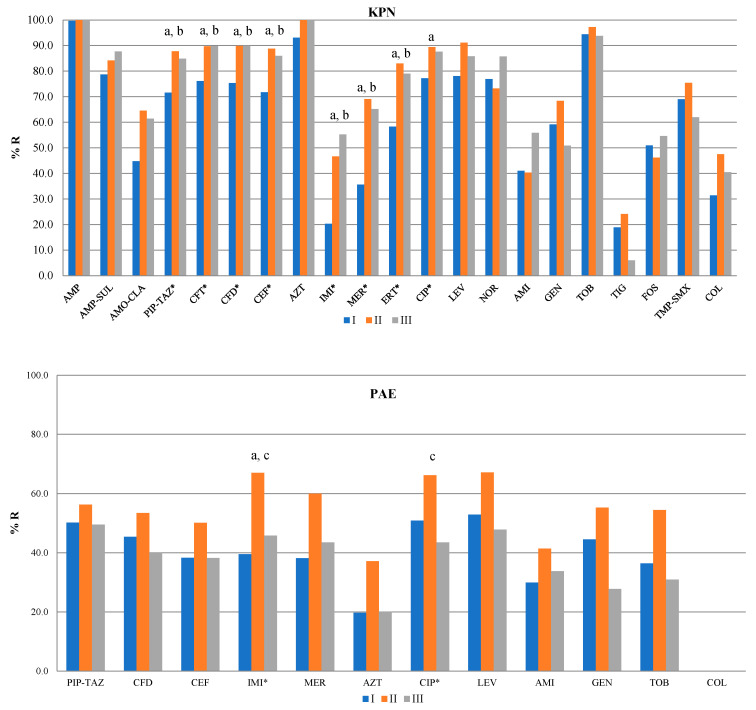

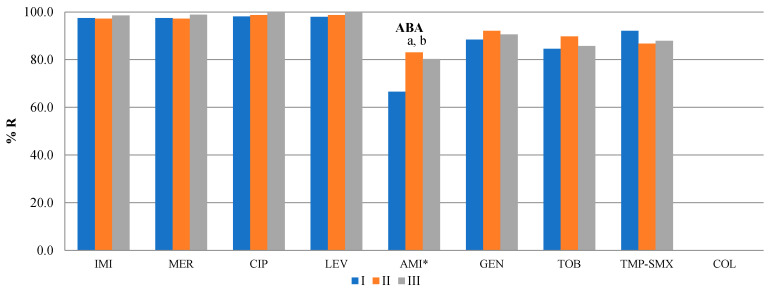
Profiles of antibiotic resistance of KPN, PAE, and ABA. *—*p* < 0.05. AMP—ampicillin, AMP-SUL—ampicillin–sulbactam, AMO-CLA—amoxicillin–clavulanic acid, PIP-TAZ—piperacillin–tazobactam, CFT—ceftriaxone, CFD—ceftazidime, CEF—cefepime, AZT—aztreonam, IMI—imipenem, MER—meropenem, ERT—ertapenem, CIP—ciprofloxacin, LEV—levofloxacin, NOR—norfloxacin, AMI—amikacin, GEN—gentamicin, TOB—tobramycin, TIG—tigecycline, FOS—fosfomycin, TMP-SMX—trimethoprim/sulfamethoxazole, COL—colistin. a—ΔII/I, b—ΔIII/I, c—ΔIII/II.

A detailed overview of the resistance profiles and their distribution throughout the study years and months is shown in Figure 4 and Supplementary File S2.

### 2.4. Antibiotic Consumption in the Hospital During the Study Periods

**Figure 5:**
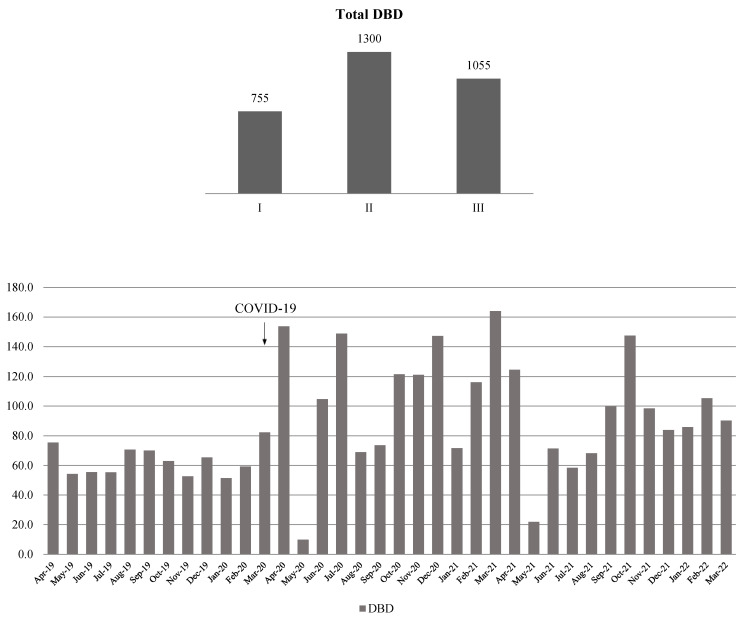
Total antibiotic consumption per year (**top**) and its distribution per month (**bottom**). There was a statistically significant increase in the antibiotic consumption in period II.

The total antibiotic consumption was 755 DBD, 1300 DBD, and 1055 DBD in periods I, II, and III, respectively. Compared to period I, there was a statistically significant increase in the antibiotic consumption in period II (*p* = 0.005) (Figure 5). A detailed overview of the statistically significant changes in the antibiotic consumption is shown in Supplementary File S3.

The most commonly used antibiotic classes for the three study periods were cephalosporins (281 DBD, 374 DBD, and 367 DBD, respectively), fluoroquinolones (82 DBD, 264 DBD, and 171 DBD, respectively), carbapenems (45 DBD, 200 DBD, and 115 DBD, respectively), nitroimidazole derivate (117 DBD, 70 DBD, and 125 DBD, respectively) and penicillins (96 DBD, 68 DBD, and 81 DBD, respectively). During period II, there was a statistically significant increase in the consumption of carbapenems (*p* < 0.001), fluoroquinolones (*p* = 0.002), polymixins (*p* = 0.001), glycylcyclins (*p* < 0.001), glycopeptides (*p* = 0.001), and oxazolidinones (*p* = 0.013) and a statistically significant decrease in the consumption of aminoglycosides (*p* = 0.001) and nitro-imidazole derivate (*p* = 0.025).

**Figure 6:**
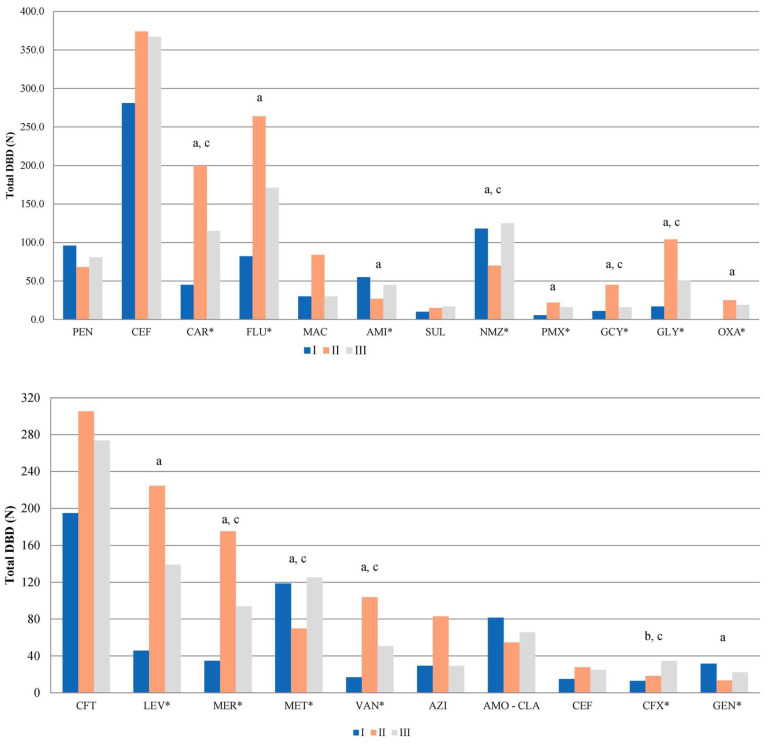
Total antibiotic consumption per antibiotic class (**top**) and per individual antibiotic (**bottom**), per study year. PEN—penicillins, CEF—cephalosporins, CAR—carbapenems, FLU—fluoroquinolones, MAC—macrolides, AMI—aminoglycosides, SUL—sulfonamides, NMZ—nitroimidazole derivates, PMX—polymyxins, GCY—glycylcyclins, GLY—glycopeptides, OXA—oxazolidinones. CFT—ceftriaxone, LEV—levofloxacin, MER—meropenem, MET—metronidazole, VAN—vancomycin, AZI—azithromycin, AMO–CLA—amoxicillin–clavulanic acid, CEF—cefepime, CFX—cefixime, GEN—gentamicin. *—*p* < 0.05. Statistically significant difference through different periods: a—ΔII/I, b—ΔIII/I, c—ΔIII/II.

During period III, compared to period II, there was a statistically significant decrease in the consumption of carbapenems (*p* = 0.031), glycylcyclins (*p* < 0.001), and glycopeptides (*p* = 0.048) and a statistically significant increase in the consumption of nitro-imidazole derivate (*p* = 0.009, Figure 6, top).

Out of the total DBD per study period, the use of antibiotics based on the AWaRe classification (access, watch, and reserve) was as follows: 40%, 58%, and 2%, respectively (period I); 17%, 76%, and 7%, respectively (period II); and 30%, 65%, and 5%, respectively (period III). There was a statistically significant increase in antibiotics from the watch and reserve groups during period II compared to period I (*p* = 0.04 and *p* = 0.035, respectively).

**Figure 7:**
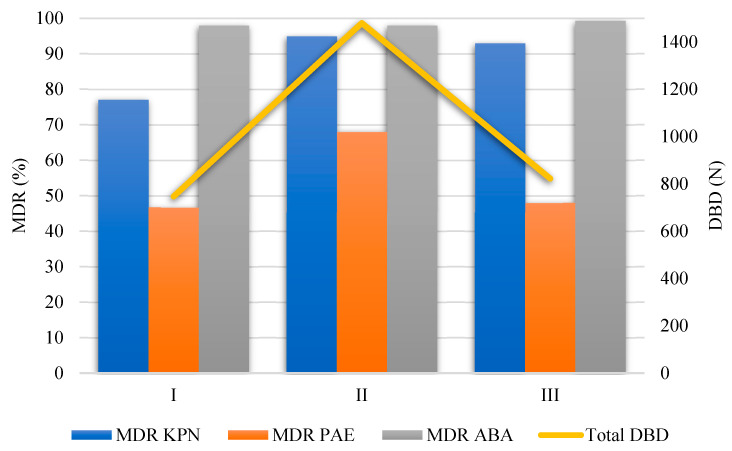
The relationship between MDR strains (%) and total DBD. Left axis—percentages (%) of the number of resistant strains. Right axis—absolute numbers (total DBD).

The most commonly used antibiotics in period I were ceftriaxone (195 DBD, 25% of the total DBD in period I), metronidazole (118 DBD, 15% of the total DBD in period I), amoxicillin–clavulanic acid (81 DBD, 10% of the total DBD in period I), levofloxacin (46 DBD, 6% of the total DBD in period I), and meropenem (34 DBD, 7.2% of the total DBD in period I). Similarly, the most commonly used antibiotics in period II were ceftriaxone (305 DBD, 23% of the total DBD in period II), levofloxacin (225 DBD, 17% of the total DBD in period II), meropenem (176 DBD, 13% of the total DBD in period II), vancomycin (104 DBD, 8% of the total DBD in period II), and azithromycin (83 DBD, 6% of the total DBD in period II). The most commonly used antibiotics in period III were ceftriaxone (276 DBD, 26% of the total DBD in period III), levofloxacin (139 DBD, 13% of the total DBD in period III), metronidazole (125 DBD, 12% of the total DBD in period III), meropenem (94 DBD, 9% of the total DBD in period III), and amoxicillin–clavulanic acid (66 DBD, 6% of the total DBD in period III) (Figure 6, bottom). Table 5 shows the top ten most commonly used antibiotics according to study periods and the relative changes during periods II and III. The relationship between MDR strains (%) and total antibiotic consumption expressed as DBD is shown in Figure 7.

### 2.5. Correlation Between Number of Patients on MV and the Occurrence of MDR

**Figure 8:**
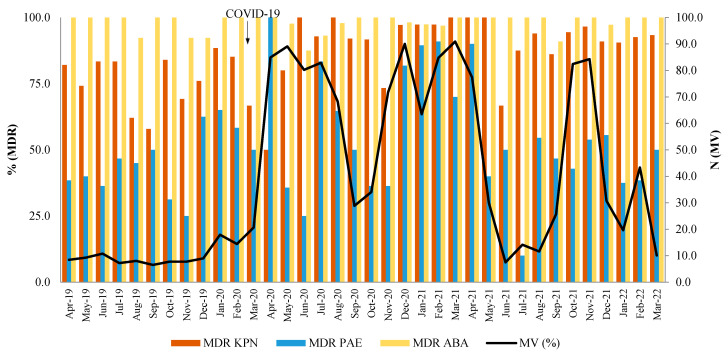
The relationship between the number of patients on mechanical ventilation (MV) and the percentage of MDR isolates.

The relationship between the number of patients on MV and the occurrence of MDR pathogens can be seen in Figure 8. There is a medium, positive correlation between the number of patients on MV and the occurrence of MDR KPN (r = 0.35, *p* = 0.009) and MDR PAE (r = 0.43, *p* = 0.009), which is not observed for MDR ABA (r = 0.09, *p* = 0.614).

## 3. Discussion

This paper investigated the impact of the COVID-19 pandemic on the prevalence and patterns of AMRs of MDR Gram-negative bacterial strains as well as the total antibiotic consumption in the tertiary healthcare center “Dr Dragisa Misovic” (Dedinje, Serbia). This center was one of the first to join the “COVID-19 system”, taking care of the most serious patients, including patients that required MV.

A total of 41,144 hospitalized patients were included in this study. Following the hospital’s entry into COVID-19 mode, there was a significant decrease in the total number of hospitalized patients, since only SARS-CoV-2-positive patients were admitted and treated, almost one-quarter of whom required an ICU admission. Nearly 75% of patients in the ICU were on MV, which may illustrate the severity of COVID-19 symptoms. However, the number of hospital admissions notably increased with the dominance of the more severe delta strain of the SARS-CoV-2 virus (June 2021, see Figure 1). This was also noted in other European countries (Slovenia, Greece, and England) [14,15,16].

The total number of bacterial isolates was lower during the pandemic, which may be surprising since MV represents a risk factor for infections but may be explained by a significant decrease in infections with polymicrobial etiology (surgical wound infections, urinary tract infections, and skin and soft tissue infections). The most common isolates before the pandemic were *E. coli*, followed by *Enterococcus* spp. and KPN, and were somewhat different during the pandemic, with dominant isolates of ABA, *Enterococcus* spp., and KPN. In this period, the number of *E. coli* isolates significantly decreased, probably due to a decrease in the number of urinary tract infections, of which *E. coli* is the most common isolate. During period III, the most prevalent isolates were *Enterococcus* spp., KPN, and *E. coli.* This is inconsistent with the reports from the European Center for Disease Prevention and Control (ECDC), where the most commonly reported bacterial isolates for the 2019–2021 period were *E. coli*, *S. aureus*, KPN, *E. faecalis*, PAE, and *E. faecium.* The biggest difference is seen in *S. aureus* isolates, which, compared to the European reports for 2020 and 2021 (about 20%), our study reported only 2–3% of the total bacterial isolates. The number of Gram-negative bacterial isolates was, on the contrary, much higher in our study, with the biggest difference observed for ABA (in European reports, 2–3% of the total bacterial isolates, while in our study, it was 9–12%) [17,18]. This pathogen distribution and frequency is, however, in line with our previous paper, where those bacterial strains were the most prevalent in both surgical and medical wards [13]. In the USA, the most common causative bacterial isolates were *Escherichia coli* (16%), *S. aureus* (11%), *Enterococcus* spp. (10%), *Klebsiella* spp. (9%), and PAE (8%), for all types of HAIs (surgical site infections, central line bloodstream infections, device-associated infections, and skin and soft tissue infections) [19]. The distribution in our results is comparable with this, but it is known that the prevalence of bacterial isolates in different hospitals may vary depending on the country/region.

The results from the Fourth National Point Prevalence Survey of 61 hospitals in Serbia are in line with the global prevalence of KPN, ABA, and PAE (16.7%, 15.2%, and 10.5%, respectively) [20]. During the COVID-19 pandemic, the frequency of infections in the hospital environment increased compared to that before the pandemic and may be responsible for the increased mortality of HAIs, mostly due to MDR-COVID-19 co-infections [21]. The global incidence of HAIs varies from 3.2% in North America to 40% in Asia, North America, and Africa [22]. In Europe, HAIs occur in 6.5% of hospitalized patients and contribute to more than 90 thousand deaths annually [23]. Along with MDR pathogens, an emerging factor for the overall outcome of HAIs and HAI-related morbidity/mortality is irrational antibiotic prescription [24]. To combat the issue of overconsumption, the WHO divided antibiotics according to their spectrum and resistance potential (AWaRe classification). This “surveillance” system may serve as a guide for more clinically rational and economically justified prescribing practices with certain positive long-term outcomes. Unfortunately, decreasing the number of MDR pathogens is still a worldwide challenge [4]. In our study, from the total number of KPN, ABA, and PAE isolates, a total of 85% were MDR strains (838/952 for KPN (88%), 795/808 for ABA (98%), and 228/431 for PAE (53%)), for a total of 1861 MDR strains out of 2211. This may be explained by the infrequent use of biocidal agents, “off-label” use of antibiotics (mild symptoms of COVID-19 pneumonia were often treated with antibiotics), increased empiric use of antibiotics, and lack of epidemiological measures to protect against resistant pathogens at the beginning of the pandemic [4].

The number of KPN isolates was high, being in the top three of the most common isolates during all three periods. This observation is supported by the corresponding EU reports, which showed an increase of more than 8% in MDR KPN strains during the pandemic (from 7% in 2019 to 16% in 2020 and 2021 of all KPN strains). In the aforementioned reports, KPN had significantly greater resistance to third-generation cephalosporins, carbapenems, fluoroquinolones, and aminoglycosides, as well as to combined treatment with third-generation cephalosporin + fluoroquinolone + aminoglycoside (92%, 62%, 89%, 78%, and 72%, respectively, of all KPN strains) [17,18,25]. Here, we confirmed the increased resistance of KPN to third-generation cephalosporin and all carbapenems. Additionally, we observed a high percentage of colistin-resistant KPN strains (30–48%). Bentivegna et al. (2021) found a significantly higher incidence of MDR bacterial infections in COVID-19 departments compared with other medical departments (29% and 19%, respectively), with extended-spectrum *β*-lactamase KPN presenting the highest increase [26,27].

The number of ABA isolates significantly increased during the pandemic (2020, 2021), which also supports the reports from the Antimicrobial Resistance Surveillance in Europe. In these EU reports for 2019, 2020, and 2021, an increase of more than 15% in MDR ABA strains was observed (from 44% in 2019 to 57% in 2020 and 67% in 2021 of all ABA isolates) [17,18,25]. Interestingly, the number of MDR ABA strains analyzed in this paper is much greater than that in the EU reports (from 98% in 2019 and 2020 to 99% in 2021 of all ABA isolates) and other countries’ national data (for example, Romania and Egypt) [28,29]. Additionally, the resistance profiles of the MDR ABA differed significantly between our results and those reported elsewhere. Here, almost all strains of ABA were resistant to imipenem, meropenem, ciprofloxacin, and levofloxacin; 85–95% of all ABA strains were resistant to gentamicin, tobramycin, and trimethoprim–sulfamethoxazole; and 65% of all ABA strains were resistant to amikacin. These findings are in line with the other national study that reported a similar resistance profile, with the lowest resistance rate for amikacin (86%) in contrast with all other antibiotics (>92%). During the pandemic, there was increased resistance of ABA strains to amikacin, varying from 80 to 95% for periods II and III, respectively. A large multicenter cross-sectional study from Egypt supports these results, with an increase of more than 20% of the amikacin-resistant strains during the pandemic [29]. Furthermore, all isolated ABA strains in our study (n = 808) were sensitive to colistin. This is somewhat different from the results of the meta-analysis that found an increase in the prevalence of colistin resistance among ABA isolates from 2% to 5% for the period 2000–2023 [30]. The highest resistance to colistin was noted in Western Europe (7%), while Eastern Europe had the lowest resistance (1%). As for specific countries, the most striking number of colistin-resistant ABA isolates was noted in Iraq (19%) and Greece (18%). Other Balkan countries were also included in this report, such as Croatia (0%) and Bosnia and Herzegovina (1%), but data for Serbia are not included. These regional data were supported by Sokolovic et al. (2023), who showed that in 2019–2022 in a single center from Bosnia and Herzegovina, resistance to colistin was 0% [31]. Colistin belongs to the reserve group of antibiotics (AWaRe) and is used as a second-line antibiotic for the treatment of the most severe infections. Therefore, its use is considered exceptional in cases of resistance to the first-line antibiotics. The fact that 100% of the ABA strains showed sensitivity to colistin is encouraging but must be maintained by proper and rational antibiotic use.

PAE is no longer at the top of the WHO’s list of priority bacterial pathogens, according to the latest report from 2024 [32]. For a long time, PAE has not been a worrisome pathogen in the hospital environment, primarily due to the favorable profile of resistance to antibiotics but also due to, as shown in this study, the decreasing number of isolates in the hospital environment. This can be explained by biological competition between ABA and PAE, as they both colonize similar environments within a host, such as the respiratory tract, and they compete for limited resources and nutrients. Both are opportunistic pathogens, particularly in hospital settings, and may compete for resources and niches within a host. However, the COVID-19 pandemic did affect its resistance profile to carbapenems, since increased resistance was observed for imipenem. This is consistent with the data from different countries (Brazil, Italy, and Ethiopia) [33,34,35]. Additionally, a study from China reported carbapenem-resistant PAE as an independent risk factor for the increased mortality during the pandemic [36].

Data related to the consumption of antibiotics during the pandemic are controversial. The latest report from the WHO shows extensive overuse of antibiotics during the COVID-19 pandemic worldwide, with a range of 33% of hospitalized patients with COVID-19 in the Western Pacific to 83% in the Eastern Mediterranean and Africa [37]. Another global report of pharmaceutical sales reported a positive association with a 10% increase in monthly COVID-19 cases and 0.8%, 1.3%, and 1.5% higher macrolide sales in Europe, North America, and Africa, respectively [38]. The pandemic has also caused AMR to increase by 15% in the USA, with 29,400 additional deaths, 40% of which were HAIs [39]. In Serbia, studies have reported antibiotic use in 72% of hospitalized adults and 47% of children with COVID-19 infection [40,41]. The newest national study of Serbia evaluating differences in antibiotic consumption rates over the past 16 years (2006–2021) revealed substantial trends in the increase in watch (ceftazidime, ceftriaxone, cefixime, ertapenem, levofloxacin, norfloxacin) and reserve (linezolid, tigecycline) groups of antibiotics, which were the most abundant during the pandemic [42]. Data from the EU/EEA ESAC-net study, which included 29 EU countries, showed that the outpatient consumption of antibiotics expressed in DDD/1000 inhabitants decreased in 2020 compared to 2019, and only in Bulgaria did this consumption increase [43]. A study by Andrews et al. conducted in primary and secondary health care institutions in 2020 showed that the total consumption of antibiotics in hospitals decreased in 2020 compared to 2015–2019, but the consumption of antibiotics prescribed for respiratory infections and broad-spectrum antibiotics increased [44]. The systemic analysis of 130 articles (mostly from the UK, India, Italy, and China) confirmed a tremendous increase in antibiotic consumption in 2020 and 2021, mostly of cephalosporins (30.1% of patients), followed by azithromycin (26% of patients) [45]. Additionally, in Spain, significantly fewer antibiotics were prescribed (−23.73%) in 2020 than in 2019, with the main reduction in March 2020 (−13.7%) and May 2020 (−42.64%) compared to the respective months in 2019 (with amoxicillin–clavulanic acid, amoxicillin, cefuroxime, and levofloxacin being the most notably decreased). The exception was the higher use of azithromycin in March 2020 compared to the same month in 2019 (+10.1%) [46]. A decrease in antibiotic consumption during the pandemic was also observed in Asia (Saudi Arabia), while an increase was observed in Australia (Vanuatu), South America (Brazil), and Asia (Lebanon, India) [47,48,49,50]. Here, we showed a significant increase in antibiotic consumption during 2020 (+70%) and 2021 (+37%). The highest increase in the overall antibiotic consumption was observed during the peaks of the pandemic, when the mortality in Serbia was the highest (December 2020, April 2021, November 2021, and February 2022). A study conducted in New York showed that as many as 79% of patients included in the study were exposed to antibiotics before being tested for SARS-CoV-2, and as many as 98% were exposed to antibiotics at some point during hospitalization [51]. Here, during the pandemic year (period II), the total DBD was 1300, which is worryingly high.

As for the evolution of different guidelines, several of them have operated worldwide since the onset of the pandemic, and this, as we suggested, may have increased non-rational antibiotic use [52]. Combination therapy with azithromycin and hydroxychloroquine has been recommended in COVID-19-positive patients by Gautret et al. (2020) in some countries despite its risk of prolonging the QTc interval [53]. Initially in the UK, empirical oral administration of doxycycline was suggested in patients who were at increased risk of COVID-19-associated complications [54]. Later, antibiotic treatment was limited to confirmed bacterial co-infections (27 May 2020, WHO) [55,56]. All this strongly influenced the high consumption of azithromycin and levofloxacin at the beginning of 2020. Additionally, for the elderly and patients in social institutions, the antibiotic of choice was amoxicillin + clavulanic acid [56], but the consumption of this antibiotic was decreased in our study.

In a study by Karulli et al. (2021), fifty percent of COVID-19 patients in the ICU developed MDR infections, the most common of which were carbapenem-resistant KPN and carbapenem-resistant ABA [57]. In India, of 21,556 patients admitted to the ICUs, 14% developed ABA, 14% PAE, and 4% KPN, while 67% of them developed *S. aureus* infection [58]. Data collected from patients with respiratory infections in the ICUs (an international cohort of hospitals from 56 different countries as a part of the SMART Surveillance Program) were in line with these data. The prevalence of KPN, ABA, and PAE isolates in the ICUs is high in Eastern Europe, Latin America, and Asia, in contrast with the United States, Canada, and Western Europe (1–3%) [59]. The high regional prevalence of these pathogens may be due to the inappropriate use of methods to prevent SARS-CoV-2 viral contamination [60]. Temperoni et al. (2021) found a greater proportion of MDR strains among Gram-negative than among Gram-positive bacteria in COVID-19-positive patients on MV, with the emphasis on carbapenem-resistant ABA [61]. Another study, conducted by Novovic et al. (2023), highlighted the risk of developing MDR ABA during the COVID-19 pandemic, especially in patients on MV [62]. In our study, MV as an independent factor was moderately/strongly correlated with the increase in resistance of MDR KPN and MDR PAE but not for MDR ABA, which is understandable given the continuous maximum resistance of ABA during the study period.

The COVID-19 pandemic had an impact on the increased incidence of candidemia [63]. Here, in periods II and III, there was a statistically significant increase in the number of *Candida* sp. isolates. Recent studies, including Brazilian data, also show a higher incidence of candidemia during the pandemic, suggesting that specific risk factors may be involved in this complication [64]. For instance, these patients had fewer candidemia-related risk factors, such as surgeries, but they had more acute risk factors linked to COVID-19 care, including immunosuppressive medications and the use of invasive devices [65].

The extensive use of antibiotics during the pandemic may increase the prevalence of *Clostridium difficile* infection, but this was not the case here. It is quite unexpected, considering the abundance of risk factors for this infection among hospitalized patients: the use of antibiotics, advanced age, prolonged hospitalization, the use of proton pump inhibitors, active immunosuppression, and comorbidities (chronic kidney disease and diabetes) [66]. However, the stable incidence of *Clostridium difficile* infection may be due to enhanced general and personal hygiene, as noted in some reports [67].

### Strengths and Limitations

This was a single-center study, with data obtained from the third-largest tertiary health-care center in the capital of Serbia. This hospital was included in the COVID-19 regime shortly after the disease outbreak, and by the end of the pandemic, it had been treating the highest number of patients from Serbia. Therefore, this may limit the applicability of these results to hospitals in other regions in Serbia with different pandemic responses and internal drug prescription guidelines/practices. Additional concerns may be the potential underreporting of MDR isolates during overwhelmed pandemic periods, a lack of genotyping for resistance mechanisms, limited generalizability beyond one institution, the unknown percentage of patients with pre-admission antibiotic use, the lack of socio-demographic and clinical characteristics of patients (e.g., age, BMI, initial symptoms, co-morbidities, and chronic pharmacotherapy), and other risk factors. These may redirect our future work to individual patient characteristics, molecular surveillance (available from 2023 for this hospital), and the effects of local stewardship interventions.

## 4. Materials and Methods

### 4.1. Study Setting

This retrospective study was conducted at the institution of a tertiary-level health care facility, University Hospital Center “Dr. Dragisa Misovic-Dedinje”, Belgrade, Serbia. This hospital has 546 hospital beds and represents the third largest tertiary-level health care hospital in Serbia. The hospital consists of the following departments: general surgery, urology, cardiology, endocrinology, gastroenterology, pulmonology, hematology, psychiatry, Ear-Nose-Throat—ENT, gynecology and obstetrics, neurology, geriatric, and pediatrics (unit specialized for children’s respiratory diseases). These departments are distributed in several separate buildings. The work of the hospital is organized through inpatient and outpatient health care and day hospitals in the internal and pediatric wards.

### 4.2. COVID-19 Pandemic in Serbia

By 28 July 2024, the COVID-19 pandemic affected more than 2.6 million people in Serbia and caused more than 18,000 deaths [1]. With a mortality rate of 0.7%, Serbia was ranked 38th place on a global list of 238 countries.

The first suspected case was confirmed on 6 March 2020. The government’s action was immediate, and the first patients were taken care of at the referent national clinic for infectious and tropical diseases. However, shortly after, many existing hospitals began to work in the COVID-19 regime, while three new COVID-19 hospitals, intended only for COVID-19 patients, were built.

### 4.3. COVID-19 Pandemic in the University Hospital Center “Dr Dragisa Misovic–Dedinje”

The University Hospital Center “Dr Dragisa Misovic-Dedinje” (Belgrade, Serbia) has been one of the most important centers for treating exclusively SARS-CoV-2-positive patients in all the pandemics. The first outpatient health care session, which was provided by health workers at this hospital, was performed continuously beginning on 19 March 2020. However, the hospital officially entered the COVID-19 regimen on 1 April 2020, so this was considered the nominal point of this study. To meet the study aims, we covered one full year before and two full years after the nominal point; thus, the overall study periods were divided as follows:

- Pre-pandemic (1 April 2019–31 March 2020—period I);
- COVID-19 pandemic (1 April 2020–31 March 2021—period II);
- COVID-19 pandemic, second year (1 April 2021–31 March 2022—period III).

During the study period, all departments of the hospital worked in the COVID-19 regime and were considered samples for this study. Shortly (in May 2020), the hospital was partially closed to COVID-19-positive patients. This was due to the systemic reorganization, pressure on the health system, and the need to reduce the intensity of the COVID-19 regime. The hospital stayed in the COVID-19 regime until 4 December 2021, when the last COVID-19-positive patients were discharged. After that, the hospital continued to treat regular (‘pre-pandemic’) pathology until the end of the study period (1 April 2022).

### 4.4. Antibiotic Susceptibility Testing

Patients with positive cultures of KPN, PAE, and ABA were included in the antimicrobial resistance analysis. The determination of the sensitivity of bacteria to antibiotics was performed in the institution’s microbiology laboratory for all patients requiring antibiotic therapy, with the exception of those for whom antibiotics were used as prophylaxis. Based on the source, all specimens were divided into the following: 1° blood, 2° respiratory tract (tracheal aspirate, bronchoalveolar lavage fluid, throat, sputum), 3° central-line catheter, 4° urine, 5° urinary catheter, 6° skin and soft tissue, and 6° other (peritoneal fluid, drainage sample, bioptate, bile, incisions, fistulas, abscesses). After the isolation of pure bacterial strains from the samples, an antibiotic sensitivity test was performed for each individual isolate with the automated Vitek^®^ 2 COMPACT (bioMerieux, Marcy l’Etoile, France), which allows both identification and antibiotic sensitivity testing or by the classic method of identification using biochemical tests and determination of sensitivity to antibiotics by the disc diffusion method. Colistin susceptibility testing was conducted by broth microdilution. For patients from whom several isolates were isolated during hospitalization, only the first isolates were analyzed. The sensitivity of bacteria to antibiotics was expressed as the MIC90. *Clostridium difficile* infection was diagnosed in patients based on a history of previous hospitalization, i.e., antibiotic therapy, who subsequently developed diarrhea; the VIDAS^®^ test for toxins A and B (CDAB), which is based on the enzyme-linked fluorescence assay (ELFA) technique (bioMereiux), was used as a confirmatory test.

The World Health Organization’s WHONET 5.6 program (versions 5.6.7, 5.6.8, and 5.6.9) installed within the institution was used for antibiotic sensitivity analysis, the purpose of which is to monitor the resistance of microorganisms to antibiotics, which, among other things, enables insight into the presence of MDR in individual departments at any time. Cutoff values of reduced sensitivity were defined according to the European Committee on Antimicrobial Susceptibility Testing (EUCAST for 2019, 2020, and 2021), and in cases where such limit values were not available, the recommendations from the Clinical and Laboratory Standards Institute (CLSI) were applied.

Multidrug resistance (MDR) was defined as an isolate resistant to at least one antibiotic from three or more antibiotic classes. The antibiotics used for all three bacterial strains (KPN, PAE, and ABA) included eight antibiotics from four drug classes: aminoglycosides (amikacin, gentamicin, and tobramycin), carbapenems (imipenem and meropenem), fluoroquinolones (ciprofloxacin and levofloxacin), and polymyxins (colistin). The antibiograms for all the KPN and PAE strains additionally included beta-lactam compounds (piperacillin–tazobactam, ceftazidime, cefepime, and aztreonam). The antibiograms for KPN additionally included beta-lactams (ampicillin, ampicillin–sulbactam, amoxicillin–clavulanic acid, and ceftriaxone), sulfonamides (trimethoprim–sulfamethoxazole), and others (fosfomycin and tigecycline). The antibiograms for ABA additionally included sulfonamides (trimethoprim–sulfamethoxazole).

The burden of resistance for each antibiotic was calculated as the proportion of all resistant isolates in relation to all KPN, PAE, and ABA isolates from all patients. All patients (including newborns and children) with microbiologically proven infection were included. If the same bacterial strain was cultured from the same patient within a 15-day interval, that sample was not included, as that was considered a repeated sample. Before entering the ICU, all patients were initially sampled, and all positive cultures were saved in a separate database (surveillance cultures). Those cultures were not included in this study.

### 4.5. Antibiotic Consumption

Antibiotic consumption data were obtained from the institution’s information system. The ATC classification of drugs according to the WHO was used for drug classification, and the consumption of antimicrobial drugs in the observed departments was represented by the number of defined daily doses (DDD) calculated per 100 bed–days (DBD). The calculation was as follows: DBD = total antibiotic consumption (mg) × 100/(DDD × total number of beds × bed occupancy index × period (days)). This study included all parenteral and oral inpatient antibiotics taken during the defined period. The obtained values were analyzed at one-month intervals during the duration of the study. The number of hospital beds in the ICU for the periods 1 April 2019–31 December 2019, 1 January 2020–1 July 2020, and 1 July 2020–1 April 2022 were 33, 83, and 184, respectively. The antibacterial drugs used for systemic use (J01), antibiotics used to treat intestinal infections (A07AA), antiprotozoal drugs, and nitro-imidazole derivatives (P01AB) were included. Antimicrobial drugs for local application on the skin and antiviral and antifungal drugs were excluded. The consumption of antibiotics is also shown according to the valid AWaRe classification (A—access, Wa—watch, Re—reserve).

### 4.6. Ethics

This research was approved by the institution’s Ethics Committee (Ethical Approval number: 1322/VII-9). Patient data were completely anonymous, and the study was a quality control project; therefore, the Ethics Committee decided that informed consent was not needed.

### 4.7. Statistical Analysis

Measures of descriptive statistics have been used: measures of central tendency (the arithmetic mean with 95% confidence interval of the mean), measures of variability (standard deviation—Std.), and indicators of structure (relative numbers). Data are presented as the mean ± Std. A t-test for independent samples was used to compare two groups of variables. Main hypotheses (the difference between the groups) were tested by using analysis of variance (ANOVA) with post hoc analysis (Bonferroni) at the level of statistical significance of *p* = 0.05. The linear Pearson correlation coefficient was used to test correlations between the variables. The strength of correlations was analyzed as follows: (0.0 < 0.1—no correlation, 0.1 < 0.3—low correlation, 0.3 < 0.5—medium correlation, 0.5 < 0.7–high correlation, 0.7 < 1—very high correlation). The software package IBM SPSS Statistics, version 22 (IBM Corp., Armonk, NY, USA), was used.

## 5. Conclusions

The COVID-19 pandemic led to an increase in antibiotic consumption (mostly fluoroquinolones and carbapenems), with ceftriaxone being the most commonly used antibiotic. There was a particularly significant increase in the consumption of reserve antibiotics.

An increase in the antibiotic resistance of KPN, PAE, and ABA was noted. The most prominent increase in resistance for KPN was for third-generation cephalosporins and carbapenems, for PAE for imipenem, and for ABA for amikacin. The pandemic also influenced the increase in the total number of ABA isolates and MDR KPN. The number of patients on MV positively correlated with the increase in the AMR of KPN and PAE. Despite higher antibiotic use, the prevalence of *C. difficile* colitis remained unchanged compared to pre-pandemic, in contrast to the higher prevalence of candidemia, which can also be attributed to the excessive use of antibiotics.

The strength of this paper is in the high number of patients and bacterial isolates. These results may indicate the need for continued and intensified implementation of measures to combat AMR, given the unfavorable predictions and increased mortality from MDR pathogens.

## Figures and Tables

**Table 1:** Number of patients included in the study periods.

Patients/Periods	I N (%)	II N (%)	III N (%)	*p* Δ II/I	*p* Δ III/I
No. of patients (total)	18,815	8927	13,402	*p* < 0.001	*p* < 0.05
No. of patients (ICU) (%)	4603 (24.5)	2043 (22.9)	3095 (23.1)	*p* < 0.001	*p* < 0.001
No. of patient on MV (%)	485 (10.7)	1424 (72.4)	1183 (36.4)	*p* = 0.002	*p* > 0.05
Average days of hospital stay	5.8	7.6	6.1	*p* < 0.05	*p* > 0.05
Average days of hospital stay (ICU)	35	9.5	21.5	*p* < 0.05	*p* > 0.05

**Table 2:** Main indications for antimicrobial therapy during the study intervals.

Indications/Periods	I N (%)	II N (%)	III N (%)	*p* ΔII/I	*p* ΔIII/I
Respiratory tract infections *	5190 (45)	6478 (47.6)	5149 (31.1)	*p* > 0.05	*p* > 0.05
Surgical wound infections	556 (4.7)	54 (0.4)	266 (1.6)	*p* < 0.05	*p* > 0.05
Sepsis/bacteremia	59 (0.5)	23 (0.2)	39 (0.2)	*p* > 0.05	*p* > 0.05
Urinary tract infections	4648 (40.1)	1260 (9.3)	1914 (11.6)	*p* < 0.05	*p* < 0.05
Skin and soft tissue infections	1050 (9.1)	586 (4.3)	965 (5.7)	*p* < 0.05	*p* > 0.05
COVID-19, virus identified	0 (0)	5137 (37.7)	8095 (49)	*p* < 0.001	*p* < 0.001
*C. difficile* infections	67 (0.6)	69 (0.5)	120 (0.8)	*p* > 0.05	*p* < 0.05
Total	11,570 (100)	13,607 (100)	16,548 (100)	*p* > 0.05	*p* < 0.05

* both upper (J00–J06) and lower (J12–J22) respiratory tract infections. A patient may have multiple diagnoses.

**Table 3:** Types and frequency of the bacterial strains during the study periods.

Isolates/Periods	I N (%)	II N (%)	III N (%)	*p* ΔII/I	*p* ΔIII/I
*Escherichia coli*	612 (18.2)	174 (6.2)	361 (11.6)	*p* < 0.05	*p* > 0.05
*Klebsiella pneumoniae (KPN)*	321 (9.5)	267 (9.6)	369 (11.8)	*p* > 0.05	*p* > 0.05
*Acinetobacter baumannii (ABA)*	163 (4.8)	357 (12.9)	288 (9.2)	*p* = 0.021	*p* > 0.05
*Pseudomonas aeruginosa (PAE)*	199 (5.9)	121 (4.4)	111 (3.6)	*p* = 0.003	*p* = 0.001
*Staphylococcus aureus*	164 (4.9)	61 (2.2)	96 (3.1)	*p* < 0.05	*p* > 0.05
*Staphylococcus epidermidis*	97 (2.8)	174 (6.2)	103 (3.3)	*p* < 0.05	*p* > 0.05
*Proteus mirabilis*	181 (5.4)	110 (3.9)	133 (4.3)	*p* < 0.05	*p* > 0.05
*Enterococcus faecalis*	294 (8.7)	160 (5.7)	142 (4.5)	*p* > 0.05	*p* < 0.05
*Enterococcus faecium*	77 (2.3)	130 (4.6)	115 (3.6)	*p* < 0.05	*p* > 0.05
*Enterococcus* spp. *	372 (11.1)	310 (11.2)	475 (15.2)	*p* > 0.05	*p* > 0.05
*Enterobacter* spp.	94 (2.8)	27 (0.9)	44 (1.4)	*p* < 0.05	*p* < 0.05
*Streptococcus* spp.	172 (5.1)	121 (4.3)	62 (2)	*p* < 0.05	*p* < 0.05
*Candida* spp. **	49 (1.4)	286 (10.3)	249 (7.9)	*p* < 0.001	*p* < 0.001
Others ***	563 (16.8)	474 (17.1)	568 (18.2)	*p* > 0.05	*p* > 0.05
No. of total isolates	3358 (100)	2772 (100)	3116 (100)	*p* > 0.05	*p* > 0.05

*—Enterococcus spp. –not classified. **—*C. albicans*, *C. glabrata*, *C. kruzei*, *C. tropicalis*, *C. parapsilosis, C. lusitaniae.* ***—Refers to species whose total number of isolates per study period was below 20.

**Table 4:** The distribution of KPN, ABA, and PAE isolates based on the site of infection and specimen type.

	KPN			ABA			PAE		
Site of Infection	I N (%)	II N (%)	III N (%)	I N (%)	II N (%)	III N (%)	I N (%)	II N (%)	III N (%)
BSI	17 (5)	32 (12)	42 (11)	16 (10)	78 (22)	66 (23)	11 (6)	7 (6)	10 (9)
RTI	12 (4)	23 (9)	14 (4)	18 (11)	25 (7)	13 (4)	19 (9)	21 (17)	6 (6)
UTI	201 (63)	59 (22)	125 (34)	32 (20)	17 (5)	25 (9)	74 (37)	18 (15)	22 (20)
CLABSI	2 (1)	17 (6)	15 (4)	3 (2)	34 (10)	12 (4)	4 (2)	3 (3)	5 (5)
CAUTI	13 (4)	112 (42)	94 (25)	29 (18)	180 (50)	128 (44)	20 (10)	49 (41)	16 (14)
SSTI	52 (16)	21 (8)	55 (15)	54 (33)	16 (5)	37 (13)	51 (25)	11 (9)	38 (34)
Other infections *	25 (8)	3 (1)	24 (6)	11 (7)	6 (2)	8 (3)	21 (11)	11 (9)	14 (12)
Total	321 (100)	267 (100)	369 (100)	163 (100)	357 (100)	288 (100)	199 (100)	121 (100)	111 (100)

BSI—bloodstream infections: blood; RTI—respiratory tract infections: tracheal aspirate, bronchoalveolar lavage fluid, throat, sputum; UTI—urinary tract infections: urine; CLABSI—central line-associated bloodstream infections: central-line catheter; CAUTI—catheter-associated urinary tract infections: urinary catheter; SSTI—skin and soft tissue infections: skin and wound; *—Other infections: peritoneal fluid, drainage sample, bioptate, bile, incisions, fistulas, abscesses. I–III—study periods.

**Table 5:** The most commonly used antibiotics during the study years (comparison to period I).

Rank	I	II	III
1.	Ceftriaxone	Ceftriaxone (+56%)	Ceftriaxone (+40%)
2.	Metronidazole	Levofloxacin (+386%)	Levofloxacin (+200%)
3.	Amoxicillin–clavulanic acid	Meropenem (+176%)	Metronidazole (+5%)
4.	Levofloxacin	Vancomycin (+511%)	Meropenem (+168%)
5.	Meropenem	Azithromycin (+186%)	Amoxicillin–clavulanic acid (−20%)
6.	Gentamicin	Metronidazole (−42%)	Vancomycin (+200%)
7.	Azithromycin	Amoxicillin–clavulanic acid (−33%)	Cefixime (+169%)
8.	Vancomycin	Cefepime (+86%)	Azithromycin (0%)
9.	Cefepime	Cefixime (+38%)	Cefepime (+66%)
10.	Cefixime	Gentamicin (−62%)	Gentamicin (−32%)

## Data Availability

The data that support the findings of this study are available from the corresponding author [M.G.] upon reasonable request.

## Supplementary Materials

The following supporting information can be downloaded at: https://www.mdpi.com/article/10.3390/antibiotics14090871/s1, Supplementary File S1. The distribution of total isolates (top) and MDR strains (bottom) of *Klebsiella pneumoniae* (KPN), *Pseudomonas aeruginosa* (PAE), and *Acinetobacter baumannii* (ABA). Supplementary File S2. Profiles of antibiotic resistance of KPN, PAE and ABA. Supplementary File S3. Total antibiotic consumption (top), consumption per antibiotic classes (middle) and per individual antibiotic (bottom), per study years.

## References

[1] (2025). World Map of Cumulative Confirmed COVID-19 Deaths per Million People.

[2] Mahoney A.R., Safaee M.M., Wuest W.M., Furst A.L. (2021). The silent pandemic: Emergent antibiotic resistances following the global response to SARS-CoV-2. iScience.

[3] Denissen J., Reyneke B., Waso-Reyneke M., Havenga B., Barnard T., Khan S., Khan W. (2022). Prevalence of ESKAPE pathogens in the environment: Antibiotic resistance status, community-acquired infection and risk to human health. Int. J. Hyg. Environ. Health.

[4] Tang K.W.K., Millar B.C., Moore J.E. (2023). Antimicrobial Resistance (AMR). Br. J. Biomed. Sci..

[5] Gonzalez-Zorn B. (2021). Antibiotic use in the COVID-19 crisis in Spain. Clin. Microbiol. Infect..

[6] Milovanović J., Jotić A., Radin Z., Ćirković I. (2021). Rational use of antibiotics during the COVID-19 pandemic. Serbian J. Med. Chamb..

[7] Rynda-Apple A., Robinson K.M., Alcorn J.F. (2015). Influenza and Bacterial Superinfection: Illuminating the Immunologic Mechanisms of Disease. Infect. Immun..

[8] Simonsen L., Spreeuwenberg P., Lustig R., Taylor R.J., Fleming D.M., Kroneman M., Van Kerkhove M.D., Mounts A.W., Paget W.J. (2013). GLaMOR Collaborating Teams. Global mortality estimates for the 2009 Influenza Pandemic from the GLaMOR project: A modeling study. PLoS Med..

[9] Abu-Rub L.I., Abdelrahman H.A., Johar A.A., Alhussain H.A., Hadi H.A., Eltai N.O. (2021). Antibiotics Prescribing in Intensive Care Settings during the COVID-19 Era: A Systematic Review. Antibiotics.

[10] Zhou F., Yu T., Du R., Fan G., Liu Y., Liu Z., Xiang J., Wang Y., Song B., Gu X. (2020). Clinical course and risk factors for mortality of adult inpatients with COVID-19 in Wuhan, China: A retrospective cohort study. Lancet.

[11] Van Laethem J., Wuyts S., Van Laere S., Koulalis J., Colman M., Moretti M., Seyler L., De Waele E., Pierard D., Lacor P. (2022). Antibiotic prescriptions in the context of suspected bacterial respiratory tract superinfections in the COVID-19 era: A retrospective quantitative analysis of antibiotic consumption and identification of antibiotic prescription drivers. Intern. Emerg. Med..

[12] Tomas A., Pavlović N., Stilinović N., Horvat O., Paut-Kusturica M., Dugandžija T., Tomić Z., Sabo A. (2021). Increase and Change in the Pattern of Antibiotic Use in Serbia (2010–2019). Antibiotics.

[13] Zivanovic V., Gojkovic-Bukarica L., Scepanovic R., Vitorovic T., Novakovic R., Milanov N., Bukumiric Z., Carevic B., Trajkovic J., Rajkovic J. (2017). Differences in antimicrobial consumption, prescribing and isolation rate of multidrug resistant Klebsiella pneumoniae, Pseudomonas aeruginosa and Acinetobacter baumannii on surgical and medical wards. PLoS ONE.

[14] Sočan M., Mrzel M., Prosenc K., Korva M., Avšič-Županc T., Poljak M., Lunar M.M., Zupanič T. (2024). Comparing COVID-19 severity in patients hospitalized for community-associated Delta, BA.1 and BA.4/5 variant infection. Front. Public Health.

[15] Karageorgou V., Papaioannou A.I., Kallieri M., Blizou M., Lampadakis S., Sfika M., Krouskos A., Papavasileiou V., Strakosha F., Vandorou K.T. (2023). Patients Hospitalized for COVID-19 in the Periods of Delta and Omicron Variant Dominance in Greece: Determinants of Severity and Mortality. J. Clin. Med..

[16] Twohig K.A., Nyberg T., Zaidi A., Thelwall S., Sinnathamby M.A., Aliabadi S., Seaman S.R., Harris R.J., Hope R., Lopez-Bernal J. (2022). Hospital admission and emergency care attendance risk for SARS-CoV-2 delta (B.1.617.2) compared with alpha (B.1.1.7) variants of concern: A cohort study. Lancet Infect. Dis..

[17] European Centre for Disease Prevention and Control (2022). Antimicrobial Resistance in the EU/EEA (EARS-Net)—Annual Epidemiological Report 2020.

[18] European Centre for Disease Prevention and Control (2022). Antimicrobial Resistance in the EU/EEA (EARS-Net)—Annual Epidemiological Report 2021.

[19] Centers for Disease Control and Prevention (2023). HAI Pathogens and Antimicrobial Resistance Report, 2018–2021.

[20] Ćirković I., Marković-Denić L., Bajčetić M., Dragovac G., Đorđević Z., Mioljević V., Urošević D., Nikolić V., Despotović A., Krtinić G. (2022). Microbiology of Healthcare-Associated Infections: Results of a Fourth National Point Prevalence Survey in Serbia. Antibiotics.

[21] Yahya R.O. (2022). Problems Associated with Co-Infection by Multidrug-Resistant Klebsiella pneumoniae in COVID-19 Patients: A Review. Healthcare.

[22] Raoofi S., PashazadehKan F., Rafiei S., Hosseinipalangi Z., NooraniMejareh Z., Khani S., Abdollahi B., SeyghalaniTalab F., Sanaei M., Zarabi F. (2023). Global prevalence of nosocomial infection: A systematic review and meta-analysis. PLoS ONE.

[23] Despotović A., Milić N., Cirković A., Milošević B., Jovanović S., Mioljević V., Obradović V., Kovačević G., Stevanović G. (2023). Incremental costs of hospital-acquired infections in COVID-19 patients in an adult intensive care unit of a tertiary hospital from a low-resource setting. Antimicrob. Resist. Infect. Control.

[24] Meybodi M.M.E., Foroushani A.R., Zolfaghari M., Abdollahi A., Alipour A., Mohammadnejad E., Mehrjardi E.Z., Seifi A. (2021). Antimicrobial resistance pattern in healthcare-associated infections: Investigation of in-hospital risk factors. Iran. J. Microbiol..

[25] European Centre for Disease Prevention and Control (2020). Antimicrobial Resistance in the EU/EEA (EARS-Net)—Annual Epidemiological Report 2019.

[26] Bentivegna E., Luciani M., Arcari L., Santino I., Simmaco M., Martelletti P. (2021). Reduction of Multidrug-Resistant (MDR) Bacterial Infections during the COVID-19 Pandemic: A Retrospective Study. Int. J. Environ. Res. Public Health.

[27] Xu L., Sun X., Ma X. (2017). Systematic review and meta-analysis of mortality of patients infected with carbapenem-resistant Klebsiella pneumoniae. Ann. Clin. Microbiol. Antimicrob..

[28] Luchian N., Olaru I., Pleșea-Condratovici A., DuceacCovrig M., Mătăsaru M., Dabija M.G., Elkan E.M., Dabija V.A., Eva L., Duceac L.D. (2025). Clinical and Epidemiological Aspects on Healthcare-Associated Infections with *Acinetobacter* spp. in a Neurosurgery Hospital in North-East Romania. Medicina.

[29] Alotaibi T., Abuhaimed A., Alshahrani M., Albdelhady A., Almubarak Y., Almasari O. (2021). Prevalence of multidrug-resistant Acinetobacter baumannii in a critical care setting: A tertiary teaching hospital experience. SAGE Open Med..

[30] Bostanghadiri N., Narimisa N., Mirshekar M., Dadgar-Zankbar L., Taki E., Navidifar T., Darban-Sarokhalil D. (2024). Prevalence of colistin resistance in clinical isolates of Acinetobacter baumannii: A systematic review and meta-analysis. Antimicrob. Resist. Infect. Control.

[31] Sokolović D., Drakul D., Vujić-Aleksić V., Joksimović B., Marić S., Nežić L. (2023). Antibiotic consumption and antimicrobial resistance in the SARS-CoV-2 pandemic: A single-center experience. Front. Pharmacol..

[32] WHO (2024). WHO Bacterial Priority Pathogens List, 2024: Bacterial Pathogens of Public Health Importance to Guide Research, Development and Strategies to Prevent and Control Antimicrobial Resistance.

[33] Mesquita G.P., Costa M.C.C., Silva M.A., Araújo L.G., Vila Nova B.G., Castro É.J.M., Castelo Branco L.C.M., Silva R.C.S.D., Marques S.G., Abreu A.G. (2023). Antimicrobial resistance of Pseudomonas aeruginosa isolated from patients with pneumonia during the COVID-19 pandemic and pre-pandemic periods in Northeast Brazil. Braz. J. Med. Biol. Res..

[34] Serretiello E., Manente R., Dell’Annunziata F., Folliero V., Iervolino D., Casolaro V., Perrella A., Santoro E., Galdiero M., Capunzo M. (2023). Antimicrobial Resistance in Pseudomonas aeruginosa before and during the COVID-19 Pandemic. Microorganisms.

[35] Araya S., Gebreyohannes Z., Tadlo G., Gessew G.T., Negesso A.E. (2023). Epidemiology and Multidrug Resistance of Pseudomonas aeruginosa and Acinetobacter baumanni Isolated from Clinical Samples in Ethiopia. Infect. Drug Resist..

[36] Zhang Y., Li Y., Zeng J., Chang Y., Han S., Zhao J., Fan Y., Xiong Z., Zou X., Wang C. (2020). Risk Factors for Mortality of Inpatients with Pseudomonas aeruginosa Bacteremia in China: Impact of Resistance Profile in the Mortality. Infect. Drug Resist..

[37] WHO (2024). WHO Reports Widespread Overuse of Antibiotics in Patients Hospitalized with COVID-19.

[38] Nandi A., Pecetta S., Bloom D.E. (2023). Global antibiotic use during the COVID-19 pandemic: Analysis of pharmaceutical sales data from 71 countries, 2020–2022. eClinicalMedicine.

[39] Sullivan C., Fisher C.R., Grabowsky L., Sertkaya A., Berlind A., Mallick S. (2025). Combating Antimicrobial Resistance During the COVID19 Pandemic: Perceived Risks and Protective Practices.

[40] Despotović A., Barać A., Cucanić T., Cucanić K., Stevanović G. (2022). Antibiotic (Mis)Use in COVID-19 Patients before and after Admission to a Tertiary Hospital in Serbia. Antibiotics.

[41] Prijić A., Gazibara T., Prijić S., Mandić-Rajčević S., Maksimović N. (2022). Factors Associated with the Antibiotic Treatment of Children Hospitalized for COVID-19 during the Lockdown in Serbia. Int. J. Environ. Res. Public Health.

[42] Filimonovic J., Ristić Z.S., Gazibara T., Saponjic V., Dotlic J., Jovanovic V., Arsovic A., Vukajlovic I., Joksimovic B., Sokolovic D. (2024). Trends and patterns of antibiotics use in Serbia from 2006 to 2021: Pre-COVID-19 period versus COVID-19 pandemic. Am. J. Infect. Control.

[43] European Centre for Disease Prevention and Control (2022). Antimicrobial Consumption in the EU/EEA (ESAC-Net)—Annual Epidemiological Report 2021.

[44] Andrews A., Budd E.L., Hendrick A., Ashiru-Oredope D., Beech E., Hopkins S., Gerver S., Muller-Pebody B. (2021). The Amu Covid-Stakeholder Group. Surveillance of Antibacterial Usage during the COVID-19 Pandemic in England, 2020. Antibiotics.

[45] Malik S.S., Mundra S. (2022). Increasing Consumption of Antibiotics during the COVID-19 Pandemic: Implications for Patient Health and Emerging Anti-Microbial Resistance. Antibiotics.

[46] Rojas-Garcia P., Antoñanzas F. (2021). Analysis of the Prescription of Antibiotics During the Implementation of COVID-19 Personal Protection Measures in a Regional Health System. Clin. Outcomes Res..

[47] Elsafi S.H., Almutairi S.H., Alsulaimani M.A., AlBahrani S., Al-Maqati T.N., Alanazi W.K., Alanazi M.N., Alamri A.A., Alkhathami M.H., Alshammari R.A. (2024). The Trend of Antibiotic Consumption After the COVID-19 Pandemic: Approach to Future Outbreaks. Infect. Drug Resist..

[48] Foxlee N.D., Lui A., Mathias A., Townell N., Lau C.L. (2022). Antibiotic Consumption in Vanuatu before and during the COVID-19 Pandemic, 2018 to 2021: An Interrupted Time Series Analysis. Trop. Med. Infect. Dis..

[49] Massarine N.C.M., de Souza G.H.A., Nunes I.B., Salomé T.M., Barbosa M.D.S., Faccin I., Rossato L., Simionatto S. (2023). How Did COVID-19 Impact the Antimicrobial Consumption and Bacterial Resistance Profiles in Brazil?. Antibiotics.

[50] Fukushige M., Ngo N.H., Lukmanto D., Fukuda S., Ohneda O. (2022). Effect of the COVID-19 pandemic on antibiotic consumption: A systematic review comparing 2019 and 2020 data. Front. Public Health.

[51] Rusic D., Vilovic M., Bukic J., Leskur D., SeseljaPerisin A., Kumric M., Martinovic D., Petric A., Modun D., Bozic J. (2021). Implications of COVID-19 Pandemic on the Emergence of Antimicrobial Resistance: Adjusting the Response to Future Outbreaks. Life.

[52] Granata G., Schiavone F., Pipitone G., Taglietti F., Petrosillo N. (2022). Antibiotics Use in COVID-19 Patients: A Systematic Literature Review. J. Clin. Med..

[53] Gautret P., Lagier J.C., Honoré S., Hoang V.T., Colson P., Raoult D. (2021). Hydroxychloroquine and azithromycin as a treatment of COVID-19: Results of an open label non-randomized clinical trial revisited. Int. J. Antimicrob. Agents.

[54] (2025). COVID-19 Rapid Guideline: Managing COVID-19.

[55] (2020). COVID-19 Rapid Guideline: Managing Symptoms (Including at the End of Life) in the Community.

[56] World Health Organization (2020). Clinical Management of COVID-19: Interim Guidance.

[57] Karruli A., Boccia F., Gagliardi M., Patauner F., Ursi M.P., Sommese P., De Rosa R., Murino P., Ruocco G., Corcione A. (2021). Multidrug-Resistant Infections and Outcome of Critically Ill Patients with Coronavirus Disease 2019: A Single Center Experience. Microb. Drug Resist..

[58] Verma V., Valsan C., Mishra P., Mund K., Dutta S., Anke G., Sasi H., Shah D. (2024). Antimicrobial Resistance Profile in ICU Patients Across India: A Multicenter, Retrospective, Observational Study. Cureus.

[59] Moise P.A., Gonzalez M., Alekseeva I., Lopez D., Akrich B., DeRyke C.A., Chen W.T., Pavia J., Palermo B., Hackel M. (2021). Collective assessment of antimicrobial susceptibility among the most common Gram-negative respiratory pathogens driving therapy in the ICU. JAC Antimicrob. Resist..

[60] Ripa M., Galli L., Poli A., Oltolini C., Spagnuolo V., Mastrangelo A., Muccini C., Monti G., De Luca G., Landoni G. (2021). COVID-BioB study group. Secondary infections in patients hospitalized with COVID-19: Incidence and predictive factors. Clin. Microbiol. Infect..

[61] Temperoni C., Caiazzo L., Barchiesi F. (2021). High Prevalence of Antibiotic Resistance among Opportunistic Pathogens Isolated from Patients with COVID-19 under Mechanical Ventilation: Results of a Single-Center Study. Antibiotics.

[62] Novović K., KuzmanovićNedeljković S., Poledica M., Nikolić G., Grujić B., Jovčić B., Kojić M., Filipić B. (2023). Virulence potential of multidrug-resistant Acinetobacter baumannii isolates from COVID-19 patients on mechanical ventilation: The first report from Serbia. Front. Microbiol..

[63] Araujo J.M., de Almeida Junior J.N., Magri M.M.C., Costa S.F., Guimarães T. (2024). Epidemiological Assessment and Risk Factors for Mortality of Bloodstream Infections by Candida sp. and the Impact of the COVID-19 Pandemic Era. J. Fungi.

[64] Lobo A.P., Cardoso-Dos-Santos A.C., Rocha M.S., Pinheiro R.S., Bremm J.M., Macário E.M., Oliveira W.K., França G.V.A. (2020). COVID-19 epidemic in Brazil: Where are we at?. Int. J. Infect. Dis..

[65] Seagle E.E., Jackson B.R., Lockhart S.R., Georgacopoulos O., Nunnally N.S., Roland J., Barter D.M., Johnston H.L., Czaja C.A., Kayalioglu H. (2022). The Landscape of CandidemiaDuring the Coronavirus Disease 2019 (COVID-19) Pandemic. Clin. Infect. Dis..

[66] Davies K., Lawrence J., Berry C., Davis G., Yu H., Cai B., Gonzalez E., Prantner I., Kurcz A., Macovei I. (2020). Risk Factors for Primary Clostridium difficile Infection; Results From the Observational Study of Risk Factors for Clostridium difficile Infection in Hospitalized Patients With Infective Diarrhea (ORCHID). Front. Public Health.

[67] Bachour S.P., Dalal R., Allegretti J.R. (2023). The impact of the COVID-19 pandemic on Clostridioides difficile infection and utilization of fecal microbiota transplantation. Ther. Adv. Gastroenterol..

