# Supplementary material for: Increased Antimicrobial Consumption, Isolation Rate, and Resistance Profiles of Multi-Drug Resistant Klebsiella pneumoniae, Pseudomonas aeruginosa, and Acinetobacter baumannii During the COVID-19 Pandemic in a Tertiary Healthcare Institution
Source: Antibiotics (Basel). 2025 Aug 29;14(9):871. doi: 10.3390/antibiotics14090871 (PMC12466518; doi:10.3390/antibiotics14090871)
Supplement: Supplementary file 1 [file antibiotics-14-00871-s001.zip › antibiotics-3767547-supplementary.pdf]

Supplementary File S1. The distribution of total isolates (top) and MDR strains (bottom) of *Klebsiella pneumoniae* (KPN), *Pseudomonas aeruginosa* (PAE), and *Acinetobacter baumannii* (ABA). Red – statistically significant increase, Blue – statistically significant decrease.

	Mean ± Std. (Total isolates)			95% Confidence interval of Mean			p		
**Total isolates (N)**	I	II	III	I	II	III	ΔII/I	ΔIII/I	ΔIII/II
KPN	26.75 ± 6.4	22.25 ± 15.08	30.75 ± 14.86	22.69 - 30.81	12.67 - 31.83	21.31 - 40.19	> 0.05	> 0.05	> 0.05
PAE	16.58 ± 4.42	10.08 ± 5.21	9.25 ± 3.62	13.77 - 19.39	6.77 - 13.4	6.95 - 11.55	.003	.001	> 0.05
ABA	13.58 ± 2.19	29.75 ± 14.54	24 ± 18.74	12.19 - 14.98	20.51 - 38.99	12.09 - 35.91	.021	> 0.05	> 0.05

	Mean ± Std. (Percentage of MDR isolates)			95% Confidence interval of Mean			p		
**MDR isolates (%)**	I	II	III	I	II	III	ΔII/I	ΔIII/I	ΔIII/II
KPN	76.02 ± 10.04	89.3 ± 14.91	89.83 ± 9.65	69.64 - 82.4	79.83 - 98.77	83.69 - 95.96	.028	.021	> 0.05
PAE	46.99 ± 10.59	69.3 ± 26.78	47.43 ± 18.19	40.26 - 53.72	52.29 - 86.31	35.88 - 58.99	.027	> 0.05	.031
ABA	98.08 ± 3.48	92.38 ± 17.61	99.01 ± 2.68	95.86 - 100.29	81.18 - 103.57	97.31 - 100.71	> 0.05	> 0.05	> 0.05

Supplementary File S2. Profiles of antibiotic resistance of KPN, PAE and ABA. AMP – ampicillin, AMP-SUL – ampicillin-sulbactam, AMO-CLA – amoxicillin-clavulanic acid, PIP-TAZ – piperacillin-tazobactam, CFT – ceftriaxone, CFD – ceftazidime, CEF – cefepime, AZT – aztreonam, IMI – imipenem, MER – meropenem, ERT – ertapenem, CIP – ciprofloxacin, LEV – levofloxacin, NOR – norfloxacin, AMI – amikacin, GEN – gentamicin, TOB – tobramycin, TIG – tigecycline, FOS – fosfomycin, TMP-SMX – trimethoprim/sulfamethoxazole, COL – colistin. Red – statistically significant increase, Blue – statistically significant decrease.

Strain/antibiotic	Mean ± Std. (% of all KPN strains)			95% Confidence interval of Mean			p		
**KPN (%)**	I	II	III	I	II	III	ΔII/I	ΔIII/I	ΔIII/II
AMP	99.68 ± 1.1	100 ± 0	100 ± 0	98.99 - 100.38	100 - 100	100 - 100	> 0.05	> 0.05	> 0.05
AMP-SUL	78.73 ± 11.9	84.13 ± 22.22	87.66 ± 10.07	71.16 - 86.29	70.01 - 98.25	81.26 - 94.06	> 0.05	> 0.05	> 0.05
AMO-CLA	44.78 ± 20.46	64.52 ± 27.13	61.45 ± 21.91	31.78 - 57.77	47.28 - 81.77	47.53 - 75.37	> 0.05	> 0.05	> 0.05
PIP-TAZ	71.63 ± 10.72	87.76 ± 14.38	84.87 ± 12.48	64.81 - 78.44	78.62 - 96.89	76.93 - 92.8	.011	.045	> 0.05
CFT	76.08 ± 9.86	89.73 ± 17.82	89.83 ± 8.41	69.82 - 82.35	78.41 - 101.06	84.48 - 95.17	.039	.037	> 0.05
CFD	75.33 ± 10.14	89.87 ± 14.68	89.95 ± 6.81	68.89 - 81.77	80.54 - 99.19	85.62 - 94.28	.008	.008	> 0.05
CEF	71.73 ± 11.33	88.82 ± 14.16	85.98 ± 13.67	64.54 - 78.93	79.82 - 97.82	77.3 - 94.67	.009	.036	> 0.05
AZT	93.12 ± 13.7	100 ± 0	100 ± 0	82.59 - 103.65	100 - 100	100 - 100	> 0.05	> 0.05	> 0.05
IMI	20.28 ± 10.07	46.65 ± 32.66	55.24 ± 24.01	13.88 - 26.68	25.9 - 67.4	39.99 - 70.49	.034	.004	> 0.05
MER	35.64 ± 15.68	69.12 ± 24.94	65.17 ± 25.38	25.68 - 45.6	53.27 - 84.96	49.04 - 81.29	.003	.033	> 0.05
ERT	58.33 ± 16.56	83 ± 20.39	79.02 ± 15.25	47.81 - 68.86	70.04 - 95.96	69.33 - 88.72	.005	.02	> 0.05
CIP	77.25 ± 11.1	89.43 ± 14.9	87.59 ± 11.34	70.19 - 84.31	79.96 - 98.89	80.39 - 94.8	> 0.05	> 0.05	> 0.05
LEV	78.04 ± 8.17	91.09 ± 15.17	85.85 ± 13.27	72.85 - 83.24	81.45 - 100.73	77.42 - 94.28	.047	> 0.05	> 0.05
NOR	76.89 ± 17.29	73.27 ± 37.92	85.7 ± 16.11	65.91 - 87.87	49.18 - 97.36	75.46 - 95.94	> 0.05	> 0.05	> 0.05
AMI	41.02 ± 14.24	40.34 ± 30.16	55.88 ± 24.6	31.97 - 50.06	21.18 - 59.5	40.24 - 71.51	> 0.05	> 0.05	> 0.05
GEN	59.13 ± 12.92	68.37 ± 24.67	50.91 ± 21.09	50.92 - 67.34	52.69 - 84.04	37.51 - 64.31	> 0.05	> 0.05	> 0.05
TOB	94.44 ± 11.78	97.23 ± 4.8	93.75 ± 15.54	85.39 - 103.5	94 - 100.45	83.88 - 103.62	> 0.05	> 0.05	> 0.05
TIG	18.88 ± 21.93	24.17 ± 30.69	6.02 ± 5.79	4.95 - 32.82	4.66 - 43.67	2.34 - 9.69	> 0.05	> 0.05	> 0.05
FOS	50.92 ± 17.6	46.14 ± 41.37	54.64 ± 22.24	39.73 - 62.1	19.85 - 72.43	40.51 - 68.77	> 0.05	> 0.05	> 0.05
TMP-SMX	69 ± 10.44	75.41 ± 17.91	61.94 ± 21.61	62.37 - 75.63	64.03 - 86.79	48.21 - 75.67	> 0.05	> 0.05	> 0.05
COL	31.42 ± 23.06	47.5 ± 42.83	40.49 ± 14.74	16.76 - 46.07	20.29 - 74.71	31.13 - 49.86	> 0.05	> 0.05	> 0.05

Strain/antibiotic	Mean ± Std. (% of all PAE strains)			95% Confidence interval of Mean			p		
**PAE (%)**	I	II	III	I	II	III	ΔII/I	ΔIII/I	ΔIII/II
PIP-TAZ	50.23 ± 15.68	56.29 ± 28.88	49.55 ± 22	40.27 - 60.2	36.89 - 75.69	34.77 - 64.32	> 0.05	> 0.05	> 0.05
CFD	45.42 ± 12.04	53.43 ± 26.71	40.01 ± 22.9	37.77 - 53.07	35.49 - 71.37	25.46 - 54.56	> 0.05	> 0.05	> 0.05
CEF	38.3 ± 13.97	50.12 ± 28.84	38.21 ± 22.09	29.42 - 47.18	30.75 - 69.49	24.17 - 52.24	> 0.05	> 0.05	> 0.05
IMI	39.5 ± 10.1	67.07 ± 26.74	45.83 ± 17.27	33.09 - 45.91	50.08 - 84.06	34.85 - 56.8	.004	> 0.05	.033
MER	38.17 ± 10.57	59.98 ± 29.7	43.49 ± 27.33	31.45 - 44.88	41.11 - 78.86	26.13 - 60.85	> 0.05	> 0.05	> 0.05
AZT	19.69 ± 19.72	37.19 ± 26.6	20.07 ± 21.14	6.44 - 32.94	19.32 - 55.06	5.87 - 34.28	> 0.05	> 0.05	> 0.05
CIP	50.92 ± 15.49	66.27 ± 26.65	43.54 ± 20.42	41.07 - 60.76	49.33 - 83.2	30.57 - 56.52	> 0.05	> 0.05	.041
LEV	52.91 ± 19.64	67.18 ± 25.68	47.82 ± 19.09	40.43 - 65.39	50.87 - 83.5	35.69 - 59.94	> 0.05	> 0.05	> 0.05
AMI	29.92 ± 13.89	41.43 ± 33.24	33.75 ± 16.08	21.09 - 38.74	20.3 - 62.55	23.53 - 43.97	> 0.05	> 0.05	> 0.05
GEN	44.52 ± 18.25	55.25 ± 36.93	27.78 ± 29.17	32.92 - 56.11	30.45 - 80.06	5.36 - 50.2	> 0.05	> 0.05	> 0.05
TOB	36.43 ± 34.3	54.45 ± 31.73	30.94 ± 22.08	14.64 - 58.22	33.13 - 75.76	16.91 - 44.97	> 0.05	> 0.05	> 0.05
COL	0 ± 0	0 ± 0	0 ± 0	0 - 0	0 - 0	0 - 0	-	-	-

Strain/antibiotic	Mean ± Std. (% of all ABA strains)			95% Confidence interval of Mean			p		
**ABA (%)**	I	II	III	I	II	III	ΔII/I	ΔIII/I	ΔIII/II
IMI	97.43 ± 3.79	97.23 ± 5.13	98.57 ± 2.92	95.02 - 99.84	93.97 - 100.48	96.71 - 100.42	> 0.05	> 0.05	> 0.05
MER	97.43 ± 3.79	97.23 ± 5.13	98.89 ± 2.66	95.02 - 99.84	93.97 - 100.48	97.2 - 100.58	> 0.05	> 0.05	> 0.05

CIP	98.08 ± 3.48	98.7 ± 3.64	100 ± 0	95.86 - 100.29	96.39 - 101.01	100 - 100	> 0.05	> 0.05	> 0.05
LEV	97.91 ± 3.8	98.7 ± 3.64	100 ± 0	95.5 - 100.32	96.39 - 101.01	100 - 100	> 0.05	> 0.05	> 0.05
AMI	66.58 ± 13.9	83.03 ± 13.4	80.29 ± 12.72	57.75 - 75.41	74.52 - 91.55	72.21 - 88.38	.015	.051	> 0.05
GEN	88.41 ± 10.26	92.06 ± 6.97	90.56 ± 9.47	81.89 - 94.93	87.63 - 96.49	84.54 - 96.58	> 0.05	> 0.05	> 0.05
TOB	84.55 ± 14.05	89.74 ± 10.08	85.74 ± 13.08	75.11 - 93.99	83.34 - 96.14	77.43 - 94.06	> 0.05	> 0.05	> 0.05
SMX-TMP	92.08 ± 10	86.66 ± 11.26	87.91 ± 10.92	85.72 - 98.43	79.5 - 93.81	80.97 - 94.85	> 0.05	> 0.05	> 0.05
COL	0 ± 0	0 ± 0	0 ± 0	0 - 0	0 - 0	0 - 0	-	-	-

Supplementary File S3. Total antibiotic consumption (top), consumption per antibiotic classes (middle) and per individual antibiotic (bottom), per study years. PEN – penicillins, CEF – cephalosporins, CAR – carbapenems, FLU – fluoroquinolones, MAC – macrolides, AMI – aminoglycosides, SUL – sulfonamides, NMZ – nitroimidazole derivates, PMX – polymyxins, GCY – glycylcyclins, GLY – glycopeptides, OXA – oxazolidinones. CFT – ceftriaxone, LEV – levofloxacin, MER – meropenem, MET – metronidazole, VAN – vancomycin, AZI – azithromycin, AMO–CLA – amoxicillin-clavulanic acid, CEF – cefepime, CFX – cefixime, GEN – gentamicin. Red – statistically significant increase, Blue – statistically significant decrease.

	Mean ± Std. (Total DBD)			95% Confidence interval of Mean			p		
Antibiotic (DBD)	I	II	III	I	II	III	ΔII/I	ΔIII/I	ΔIII/II
**Total**	62.97 ± 9.94	108.48 ± 45.25	87.97 ± 32.13	56.65 - 69.28	79.72 - 137.23	67.55 - 108.38	.005	> 0.05	> 0.05

	Mean ± Std. (DBD per antibiotic class)			95% Confidence interval of Mean			p		
**Antibiotic class**	I	II	III	I	II	III	ΔII/I	ΔIII/I	ΔIII/II
PEN	7.98 ± 2.3	5.63 ± 2.51	6.75 ± 3	6.52 - 9.44	4.03 - 7.22	4.85 - 8.66	> 0.05	> 0.05	> 0.05
CEF	23.43 ± 6.25	31.17 ± 14.03	30.64 ± 11.34	19.46 - 27.4	22.26 - 40.09	23.44 - 37.85	> 0.05	> 0.05	> 0.05
CAR	3.74 ± 0.56	16.64 ± 8.84	9.62 ± 6.49	3.39 - 4.1	11.02 - 22.26	5.49 - 13.74	<.001	> 0.05	.031
FLU	6.84 ± 1.92	22 ± 13.75	14.24 ± 10.47	5.62 - 8.06	13.26 - 30.73	7.58 - 20.89	.002	> 0.05	> 0.05
MAC	2.52 ± 3.88	6.96 ± 8.12	2.49 ± 1.53	0.05 - 4.98	1.79 - 12.12	1.52 - 3.46	> 0.05	> 0.05	> 0.05
AMI	4.87 ± 1.48	2.29 ± 1.43	3.79 ± 1.6	3.93 - 5.81	1.38 - 3.2	2.77 - 4.81	.001	> 0.05	> 0.05
SUL	0.89 ± 0.31	1.3 ± 1	1.44 ± 0.79	0.69 - 1.09	0.66 - 1.93	0.94 - 1.95	> 0.05	> 0.05	> 0.05
NMZ	9.88 ± 1.56	5.82 ± 3.81	10.44 ± 4.56	8.89 - 10.87	3.4 - 8.24	7.54 - 13.33	.025	> 0.05	.009
PMX	0.45 ± 0.15	1.82 ± 1	1.31 ± 1.12	0.35 - 0.55	1.18 - 2.45	0.6 - 2.02	.001	> 0.05	> 0.05
GCY	0.91 ± 0.27	3.74 ± 1.68	1.33 ± 0.8	0.73 - 1.08	2.67 - 4.81	0.82 - 1.83	<.001	> 0.05	<.001
GLY	1.39 ± 0.41	8.66 ± 6.72	4.23 ± 3.08	1.13 - 1.65	4.39 - 12.93	2.28 - 6.19	.001	> 0.05	.048
OXA	0.04 ± 0.08	2.09 ± 2.16	1.59 ± 1.84	-0.01 - 0.09	0.72 - 3.46	0.42 - 2.75	.013	> 0.05	> 0.05

	Mean ± Std. (DBD per antibiotic)			95% Confidence interval of Mean			p		
**Antibiotic**	I	II	III	I	II	III	ΔII/I	ΔIII/I	ΔIII/II
CFT	16.24 ± 6.71	25.44 ± 13.06	22.8 ± 8.94	11.97 - 20.5	17.15 - 33.74	17.12 - 28.48	> 0.05	> 0.05	> 0.05
LEV	3.83 ± 1.52	18.7 ± 12.41	11.6 ± 9.89	2.86 - 4.79	10.82 - 26.59	5.31 - 17.88	.001	> 0.05	> 0.05
MER	2.9 ± 0.74	14.59 ± 7.95	7.83 ± 5.36	2.43 - 3.37	9.54 - 19.64	4.42 - 11.24	<.001	> 0.05	.016
MET	9.88 ± 1.55	5.82 ± 3.81	10.44 ± 4.56	8.9 - 10.87	3.4 - 8.24	7.54 - 13.33	.025	> 0.05	.009
VAN	1.39 ± 0.41	8.66 ± 6.72	4.23 ± 3.08	1.13 - 1.65	4.39 - 12.93	2.28 - 6.19	.001	> 0.05	.048
AZI	2.45 ± 3.9	6.91 ± 8.14	2.45 ± 1.52	-0.03 - 4.92	1.74 - 12.08	1.48 - 3.41	> 0.05	> 0.05	> 0.05
AMO-CLA	6.8 ± 1.95	4.56 ± 2.13	5.48 ± 2.86	5.56 - 8.04	3.2 - 5.91	3.66 - 7.29	> 0.05	> 0.05	> 0.05
CEF	1.24 ± 0.7	2.32 ± 1.62	2.08 ± 1.02	0.8 - 1.69	1.28 - 3.35	1.43 - 2.73	> 0.05	> 0.05	> 0.05
CFX	1.09 ± 0.48	1.52 ± 0.91	2.88 ± 1.66	0.78 - 1.39	0.94 - 2.09	1.82 - 3.93	> 0.05	.001	.017
GEN	2.64 ± 0.86	1.11 ± 0.87	1.86 ± 0.94	2.09 - 3.18	0.56 - 1.66	1.26 - 2.46	.001	> 0.05	<.001
